# Using Common Spatial Distributions of Atoms to Relate Functionally Divergent Influenza Virus N10 and N11 Protein Structures to Functionally Characterized Neuraminidase Structures, Toxin Cell Entry Domains, and Non-Influenza Virus Cell Entry Domains

**DOI:** 10.1371/journal.pone.0117499

**Published:** 2015-02-23

**Authors:** Arthur Weininger, Susan Weininger

**Affiliations:** Weininger Works Incorporated, Thornhill, Ontario, Canada; Institute Pasteur, FRANCE

## Abstract

The ability to identify the functional correlates of structural and sequence variation in proteins is a critical capability. We related structures of influenza A N10 and N11 proteins that have no established function to structures of proteins with known function by identifying spatially conserved atoms. We identified atoms with common distributed spatial occupancy in PDB structures of N10 protein, N11 protein, an influenza A neuraminidase, an influenza B neuraminidase, and a bacterial neuraminidase. By superposing these spatially conserved atoms, we aligned the structures and associated molecules. We report spatially and sequence invariant residues in the aligned structures. Spatially invariant residues in the N6 and influenza B neuraminidase active sites were found in previously unidentified spatially equivalent sites in the N10 and N11 proteins. We found the corresponding secondary and tertiary structures of the aligned proteins to be largely identical despite significant sequence divergence. We found structural precedent in known non-neuraminidase structures for residues exhibiting structural and sequence divergence in the aligned structures. In N10 protein, we identified staphylococcal enterotoxin I-like domains. In N11 protein, we identified hepatitis E E2S-like domains, SARS spike protein-like domains, and toxin components shared by alpha-bungarotoxin, staphylococcal enterotoxin I, anthrax lethal factor, clostridium botulinum neurotoxin, and clostridium tetanus toxin. The presence of active site components common to the N6, influenza B, and S. *pneumoniae* neuraminidases in the N10 and N11 proteins, combined with the absence of apparent neuraminidase function, suggests that the role of neuraminidases in H17N10 and H18N11 emerging influenza A viruses may have changed. The presentation of E2S-like, SARS spike protein-like, or toxin-like domains by the N10 and N11 proteins in these emerging viruses may indicate that H17N10 and H18N11 sialidase-facilitated cell entry has been supplemented or replaced by sialidase-independent receptor binding to an expanded cell population that may include neurons and T-cells.

## Introduction

The ability to identify the functional correlates of structural and sequence variation in proteins is especially critical in evaluating functional changes in emerging pathogens and interacting pathogen systems. Pathogenic influenza A viruses have emerged with expanded tissue preferences, reassortment opportunities with other viral species, and interactions with bacterial species. An avian-origin pathogenic H7N9 influenza A virus has emerged in China that causes severe pneumonia and has adapted to replicate in the human conducting and lower airways of humans [1]. A short period of viral shedding of H5N1 HPA1 influenza A virus indicates that emergent influenzas can reinfect a population of hosts can over several transmission cycles in naive hosts [2]. Bacterial neuraminidases have been found to rescue influenza virus replication from being inhibited by the neuraminidase inhibitor zanamivir [3]. Reassortment between avian and human influenza viruses has been found to be mainly between the matrix and neuraminidase gene segments [4].

South and Central American emergent influenza A viruses H17N10, isolated from bats in Guatemala, and H18N11, isolated from bats in Peru, have highly sequence divergent N10 and N11 proteins that do not process the artificial substrate methylumbelliferyl-N-acetyl-α-D-neuraminic acid (“MUNANA”) [5,6,7]. The N10 and N11 proteins were characterized as “neuraminidase-like” because the components of a functional active site were not identified in the structural reports and the proteins showed no activity by cleavage assays, e.g., MUNANA cleavage. No other N10 or N11 protein cell entry domains were identified in the reports of the x-ray crystal structures of these proteins [5,6,7] which were deposited to the Protein Data Bank. The lack of activity of the N10 and N11 proteins is problematic as the loss of sialidase activity, in the absence of some compensating change, would be expected to reduce the fitness of any influenza A virus that incorporates these proteins.

In this study, we used neuraminidase and non-neuraminidase structures deposited to the Protein Data Bank to interpret the N10 and N11 protein structures. We used the common relative spatial occupancy of atoms in N10 and N11 proteins and functionally validated influenza A, influenza B, and bacterial neuraminidases to superpose the structures. Using the superposed structures, we identified a previously unidentified site in the N10 and N11 proteins containing conserved neuraminidase active site residues. We identified variable loop regions in the N10 and N11 proteins that present residues forming domains associated with cell entry in non-neuraminidase proteins, such as toxins and hepatitis E and SARS viral coat proteins. The absence of demonstrated neuraminidase activity with the presence of new cell entry domain components in N10 and N11 proteins suggest that N10 and N11 protein-containing viruses may enter cells without a functioning sialidase, i.e., by binding to alternative receptors such as ACE2, acetylcholine, and MHC II receptors on an expanded receptive cell population, including cells such as neurons and T-cells.

## Results

### Spatial Alignment of Structures Using Distributed Common Spatial Occupancy of Atoms

Reported structures of N10 protein [5] (“N10P”), N11 protein [7] (“N11P”), N6 neuraminidase (“N6N”) [8], influenza B neuraminidase (“IBN”) [9] and a *S*. *pneumoniae* neuraminidase (“SPN)” [10] were spatially aligned by superposition of main chain oxygen atoms in each of the five structures that we found to have common distributed geometry. The atoms listed in Table 1 were found to have nearly identical spatial distribution in the N10P, N11P, N6N, IBN, and SPN structures. As the residue numbering varies among neuraminidase structures and sequences, a Consensus Numbering System for Atoms (“CNSA”) and Residues (“CNSR”) were created to relate corresponding atoms and residues in the structures. The N10P, N11P, IBN, and SPN structures, along with any associated non-protein atoms or molecules, were rotated and translated into a common reference orientation by superposition of the corresponding CNSA118, CNSA224, and CNSA276 atoms onto N6N atoms listed in Table 1. Superposition of the corresponding CNSA atoms listed in Table 1 allowed us to examine the spatial overlap of residues of N10P and N11P with residues in structures of neuraminidases whose function was established, i.e. N6N, IBN, and SPN.

**Table 1:** Conserved atom geometry for N6N, N10P, N11P, IBN, and SPN structures.

Atom Description	PDB	Protein	Chain	Res. #	Res. Type	Atom #	Atom Type
CNSA 118: O	1W1X	N6	B	1124	ARG	3302	O
	4FVK	N10	B	118	ARG	3169	O
	4K3Y	N11	A	118	ARG	273	O
	1A4G	INB	A	115	ARG	312	O
	3H72	SPN	A	347	ARG	207	O
CNSA 224: O	1W1X	N6	B	1231	ARG	4122	O
	4FVK	N10	B	224	ARG	3974	O
	4K3Y	N11	A	224	ARG	972	O
	1A4G	INB	A	222	ARG	1160	O
	3H72	SPN	A	567	GLY	1957	O
CNSA 276: O	1W1X	N6	B	1283	GLU	4520	O
	4FVK	N10	B	276	GLU	4385	O
	4K3Y	N11	A	276	GLU	1364	O
	1A4G	INB	A	274	GLU	1559	O
	3H72	SPN	A	647	GLU	2569	O

### Structurally Identical, Structurally Corresponding, and Structurally Divergent Residues

**Fig 1:**
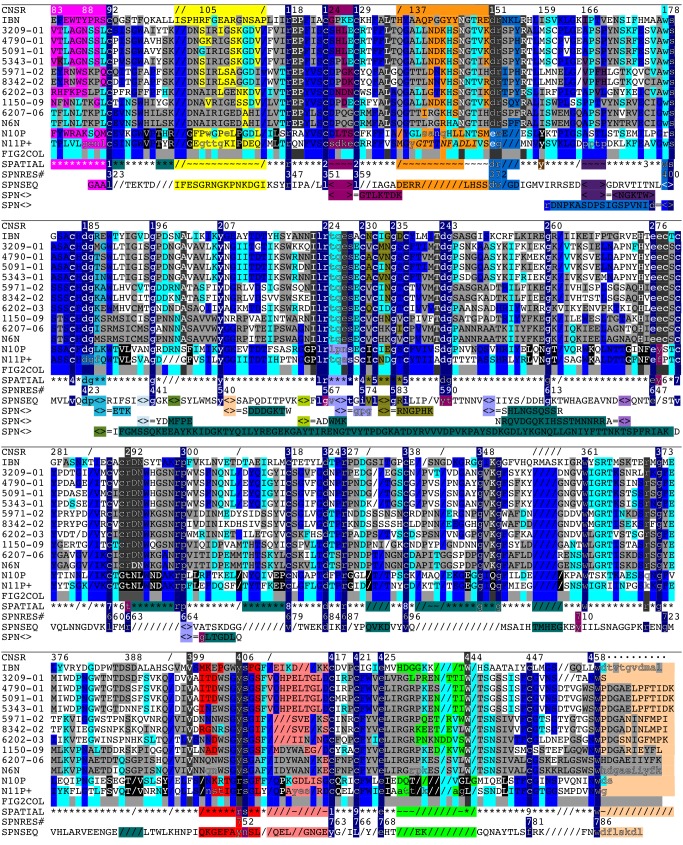
Structural alignment of N10P and N11P with influenza and bacterial neuraminidases. Sequences of N6N, N10P, N11P, IBN, and SPN are shown aligned by common spatial occupancy of residues in their superposed structures. Other influenza A sequences are shown aligned by sequence identity to the structurally aligned N6N, N10P, N11P, and INB residues. Structurally invariant residues above the 'SPATIAL' row and spatially corresponding SPN residues below the 'SPATIAL' row are shaded dark blue. If both the N10P and N11P residues are present in the rest of the column above the 'SPATIAL' row, then the corresponding residues are shaded medium blue. If either, but not both, of the N10P or N11P residue are present in the rest of the column above the 'SPATIAL' row, then the corresponding residues are shaded light blue. If the non-N10P, non-N11P residues are identical to each other but do not match either N10P or N11P, then the non-N10P, non-N11P residues are shaded dark grey. If either of the non-N10P/N11P residues match either IBN or N6P but do not match either N10 or N11, then the residue is shaded light grey. If the N10P and N11P residues are identical to each other but do not match any other residue in that column, then the N10P and N11P residues are shaded black. Upside VLR residues, not shaded as above, are shaded yellow, orange, brown, purple, red, green, and light brown, according to position in the protein and this color is also shown in that column in the 'SPATIAL' row. Residues shaded deep teal are residues in protein loops that deviate spatially from structure common to N6N, N10P, N11P, IBN, and SPN. In the 'SPNSEQ' row, "<>" means that there is an insertion of additional residues that are listed after the "<> = " in the 'SPN<>' row(s) directly below the 'SPNSEQ' row. In the 'SPATIAL' row, the symbol: "*" means spatially conserved, "/" means missing, "~" means not spatially conserved; and a number indicates corresponding cysteines in disulfide bridges. Lowercase residues represent residues shown as spheres in Figs. 4–13.

Each residue side chain in the superposed N10P and N11P structures was evaluated to determine if it had identity to, and spatial correspondence with, residues in the N6N or IBN structures. Structurally Identical Residues (“SIRs”) are the identical residues at the same relative spatial position. Structurally Corresponding Residues (“SCRs”) are different residues in structures with residue atom overlap and the same residue orientation relative to secondary structure. N6N, N10P, N11P, and IBN SIRs and corresponding SPN SIRs and SCRs are listed in Table 2 along with inter-residue offsets, i.e., the residue relative chain positions between successive entries in each column. Cysteines participating in disulfide bridges account for roughly one third (14 of the 44 (32%)) of the SIRs in N6N, N10P, N11P, and IBN shown in Fig. 1 and listed in Table 2. SIRs represent the invariant core of the influenza virus neuraminidases in the study sample that is shared by N10P and N11P.

**Table 2:** Consensus structural core residues and offsets.

N6N		N11P		N10P		IBN		SPN	
Residue	Offset	Residue	Offset	Residue	Offset	Residue	Offset	Residue	Offset
C 98	0	C 92	0	C 92	0	C 86	0	L 323	0
R 124	26	R 118	26	R 118	26	R 115	29	R 347	24
C 130	6	C 124	6	C 124	6	C 121	6	L 351	4
C 135	5	C 129	5	C 129	5	C 126	5	L 359	8
D 157	22	E 151	22	E 153	24	D 148	22	D 372	13
R 158	1	Q 152	1	R 178	25	R 149	1	R 400	28
W 185	27	W 178	26	W 154	-24	W 176	27	W 373	-27
S 186	1	S 179	1	S 179	25	S 177	1	D 417	44
C 190	4	C 183	4	C 183	4	C 181	4	V 421	4
D 192	2	D 185	2	D 185	2	D 183	2	D 423	2
G 193	1	G 186	1	G 186	1	G 184	1	P 424	1
G 203	10	G 196	10	G 196	10	G 194	10	G 441	17
Y 214	11	Y 207	11	Y 207	11	Y 205	11	Y 540	99
L 230	16	L 223	16	L 223	16	L 221	16	L 566	26
R 231	1	R 224	1	R 224	1	R 222	1	G 567	1
S 235	4	S 228	4	S 228	4	S 226	4	T 572	5
C 237	2	C 230	2	C 230	2	C 228	2	I 574	2
C 239	2	C 232	2	C 232	2	C 230	2	L 576	2
G 242	3	G 235	3	G 235	3	G 233	3	G 583	7
C 244	2	C 237	2	C 237	2	C 235	2	I 585	2
D 250	6	D 243	6	D 243	6	D 241	6	Y 590	5
G 251	1	G 244	1	G 244	1	G 242	1	T 591	1
G 267	17	G 260	16	G 260	16	G 258	16	G 613	22
E 283	16	E 276	16	E 276	16	E 274	16	E 647	34
C 287	4	C 280	4	C 280	4	C 278	4	V 650	3
C 296	9	C 289	9	C 289	9	C 288	10	L 660	10
R 299	3	T 292	3	T 292	3	R 291	3	R 663	3
R 307	8	R 300	8	R 300	8	R 299	8	-	-
P 308	1	P 301	1	P 301	1	P 300	1	G 664	1
C 325	17	C 318	17	C 318	17	C 317	17	V 679	15
D 331	6	D 324	6	D 324	6	D 323	6	D 684	5
R 334	3	R 327	3	R 327	3	R 326	3	R 687	3
C 343	9	C 338	11	C 338	11	C 336	10	V 696	9
G 355	12	G 348	10	G 348	10	G 346	10	-	-
G 358	3	G 351	3	G 351	3	G 349	3	-	-
W 368	10	W 361	10	W 361	10	W 363	14	Y 710	14
R 378	10	R 406	45	R 406	45	R 373	10	R 721	11
G 380	2	G 373	-33	G 373	-33	G 375	2	G 724	3
Y 412	32	F 277	-96	Y 277	-96	Y 408	33	Y 752	28
S 413	1	S 407	130	S 407	130	S 409	1	N 753	1
C 425	12	C 417	10	C 417	10	C 419	10	L 763	10
C 429	4	C 421	4	C 421	4	C 423	4	L 766	3
E 433	4	E 425	4	E 425	4	E 427	4	E 768	2
C 455	22	C 447	22	C 447	22	C 446	19	F 781	13
W 466	11	W 458	11	W 458	11	W 455	9	W786	5

### N10P and N11P Alignment with Influenza A, Influenza B, and Bacterial Neuraminidases

Fig. 1 shows the annotated and structurally aligned sequences of N6N, IBN, N10P, N11P, and SPN. Sequences of Influenza A N1 [11–14], N2 [15,16], N3 [17], N6 [18], and N9 [19] neuraminidases were aligned by sequence identity to the structurally aligned N6N, N10P, N11P, and IBN residues. Table 3 lists the abbreviations and descriptions of structures, sequences, and related references used in this study. Sequences used in this study are found in **S1 File**. The N11 [20] (“N11P+”) sequence was used in Fig. 1 instead of the sequence from N11P as the N11P+ sequence is identical to the N11P sequence with the exception that it contains an additional 10 residues missing from the N11P structure sequence. The use of structure to organize sequence, as illustrated in Fig. 1, provides a compact summary of structural variation and its relationship to sequence variation within a class of proteins.

**Table 3:** Abbreviations, structure sources, and sequence sources.

Abbr.	Ref.#	Sequence ID	Description
ABT	[26]	2ABX.pdb	alpha-bungarotoxin complexed to acetylcholine receptor
ALF	[27]	1YQY.pdb	anthrax lethal factor fragment
CBN	[28]	3ZUQ.pdb	clostridium botulinum neurotoxin type b
CNSR			consensus numbering system for residues (as found in 4FVK)
E2S	[23]	3RKD.pdb	hepatitis E virus E2S domain genotype I (complexed with a neutralizing Ab)
FIG2COL			coloring used in Fig. 2 spheres
IBN	[9]	1A4G.pdb	neuraminidase [Influenza B virus B/Beijing/1/87] complexed with zanamivir
N10P	[5]	4FVK.pdb	N10 protein derived from bat influenza A virus fragment
N11P	[7]	4K3Y.pdb	N11 protein of A/flat-faced bat/Peru/033/2010(H18N11)
N11P+	[7,20]	4K3Y.pdb+	4K3Y.pdb [7] with 10 missing residues 139–148 from 1259–11 [19]
N6N	[8]	1W1X.pdb	neuraminidase duck subtype N6 complex with sialic acid (NANA, NEU5AC)
SARSSP	[24]	3SCK.pdb	SARS spike protein receptor-binding domain
SEI	[22]	2G9H.pdb	staphylococcal enterotoxin I (SEI) chain D and human MHC II molecule
SPATIAL			residue correspondence and coloring used in Figs. 4–13
SPN	[10]	3H72.pdb	streptococcus pneumoniae D39 neuraminidase A precursor with NANA
SPN<>			residue loops not included in 'SPNSEQ' row
SPNSEQ			SPN sequence without loop regions
SPNRES#			SPN residue numbering
SUBP	[25]	2KS9.pdb	substance P with NK1R, tachykinin receptor 1
TTX	[29]	1DLL.pdb	receptor binding fragment H(C) of clostridium tetanus toxin
1150–09	[19]	AHA11501.1	neuraminidase [Influenza A virus (A/ZhejianG/DTID-ZJU10/2013(H7N9))]
1259–11	[20]	CY125947.1	N11 protein [Influenza A virus (A/bat/Peru/033/2010(H18N11))]
3209–01	[11]	ADR32096.1	neuraminidase [Influenza A virus (A/Lyon/1364/2007(H1N1))]
4790–01	[12]	ACJ47909.1	neuraminidase [Influenza A virus (A/environment/Qinghai/1/2008(H5N1))]
5091–01	[13]	AF509109.2	neuraminidase [Influenza A virus (A/Chicken/Hong Kong/873.3/01 (H5N1))]
5343–01	[14]	AEV53435.1	neuraminidase [Influenza A virus (A/Fukushima/09FY004/2009(H1N1))]
5971–02	[15]	ADG59718.1	neuraminidase [Influenza A virus (A/El Salvador/2-Q226L/1957(H2N2))]
6202–03	[16]	AAO62026.1	neuraminidase [Influenza A virus (A/Goose/HonGKonG/27404/78(H5N3))]
6207–06	[17]	AAO62070.1	neuraminidase [Influenza A virus (A/quail/NanchanG/4–034/2000(H4N6))]
8342–02	[18]	AGW83423.1	neuraminidase [Influenza A virus (A/Djibouti/N09200/2009(H3N2))]

### Structure Alignment Reveals Active Site Component Superposition

**Fig 2:**
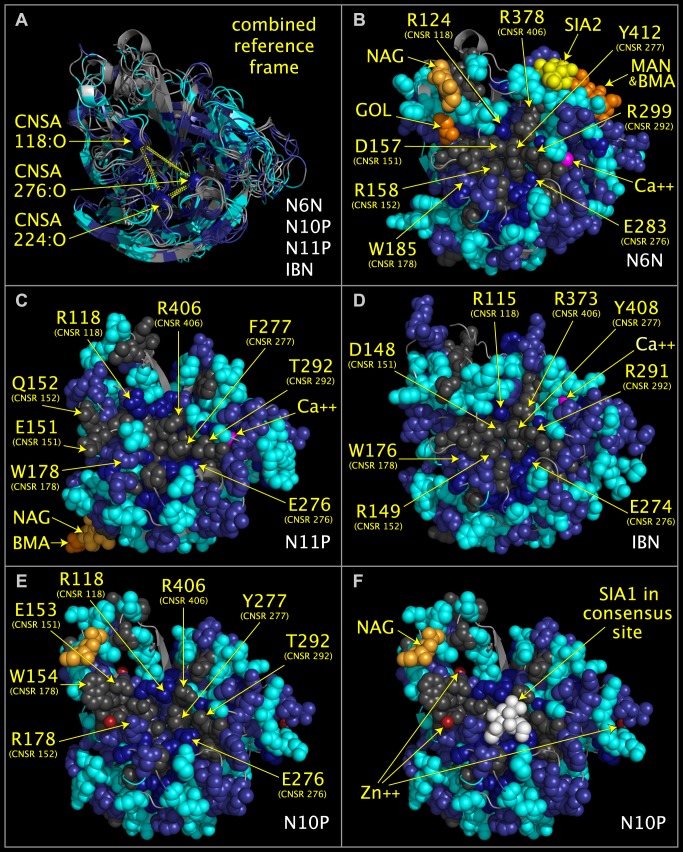
Consensus active site components of N6N, N10P, N11P, and IBN. Structure ribbon and residue spheres are color-coded at each structurally aligned position as in the 'FIG2COL' row in Fig. 1. Fig. 2A shows structure ribbons representing influenza A N10P [1], N6N [2], N11P [3], and IBN [4]) structures superposed using CNSA118:O, CNSA224:O, and CNSA276:O atoms. Fig. 2B-2F show structure ribbons and residues spheres of N6N (Fig. 2B), N11P (Fig. 2C), IBN (Fig. 2D), and N10P (Fig. 2E-2F). Fig. 2F also shows sialic acid (white spheres) positioned relative to N10P by its superposition onto N6N, which was crystalized with sialic acid in its binding pocket. Fig. 2E and 2F residues side chains in the area of the sialic acid have been repositioned slightly from the crystal structure positions to approximate the positions of the corresponding superposed N6N residues but no other side chain or main chain atoms have been moved.

Despite a high degree of sequence divergence of N10P and N11P relative to other influenza A viruses, we determined that the N10P and N11P structures have shared secondary and tertiary structure with the functionally validated sialidases, N6N and IBN. The superposed structure ribbons of N6N, N10P, N11P, and IBN, with the overlapped CNSA118, CNSA224, and CNSA oxygen atoms, are shown in Fig. 2A. Fig. 2A shows the active sites of N6N and IBN superposed with N10P and N11P regions containing similar active site components. The CNSRs shown in Fig. 2 are listed in Table 4. It can be seen by an inspection of Fig. 2B-2E that, while the positions of key residues in the protein chains have not been conserved, many of the same residues are presented from alternative loops and contribute similar components to the N10P and N11P tertiary location superposed with the N6N and IBN active sites. Fig. 2 panel E shows N10P with several side chains repositioned slightly to approximate the N6N contacts with the sialic acid. Fig. 2C and 2E show that N10P and N11P contain active site structural components common to active sialidases despite their highly divergent sequences. The superposition of N6N, with its sialic acid, and N10P provides a putative positioning of sialic acid relative to the N10P structure. This positioning of sialic acid relative to N10P is shown in Fig. 2 panel F. Structural reports [5,6,7] without benefit of common spatial occupancy alignment were unable to identify a site in either N10P or N11P where key components of functional sialidases were present in a similar geometry and in a similar tertiary position of other sialidases.

**Table 4:** Consensus Numbering System for Residues (CNSR) of selected key residues.

CNSR #	Protein	PDB	Residue #	Residue
118	N6N	1W1X	124	ARG
118	N10P	4FVK	118	ARG
118	N11P	4K3Y	118	ARG
118	IBN	1A4G	115	ARG
118	SPN	3H72	347	ARG
151	N6N	1W1X	157	ASP
151	N10P	4FVK	153	GLU
151	N11P	4K3Y	151	GLU
151	IBN	1A4G	148	ASP
151	SPN	3H72	372	ASP
152	N6N	1W1X	158	ARG
152	N10P	4FVK	178	ARG
152	N11P	4K3Y	152	GLN
152	IBN	1A4G	149	ARG
152	SPN	3H72	366	ARG
178	N6N	1W1X	185	TRP
178	N10P	4FVK	154	TRP
178	N11P	4K3Y	178	TRP
178	IBN	1A4G	176	TRP
178	SPN	3H72	373	TRP
276	N6N	1W1X	283	GLU
276	N10P	4FVK	276	GLU
276	N11P	4K3Y	276	GLU
276	IBN	1A4G	274	GLU
276	SPN	3H72	647	GLU
277	N6N	1W1X	412	TYR
277	N10P	4FVK	277	TYR
277	N11P	4K3Y	277	PHE
277	IBN	1A4G	408	TYR
277	SPN	3H72	752	TYR
292	N6N	1W1X	299	ARG
292	N10P	4FVK	292	THR
292	N11P	4K3Y	292	THR
292	IBN	1A4G	291	ARG
292	SPN	3H72	663	ARG
406	N6N	1W1X	378	ARG
406	N10P	4FVK	406	ARG
406	N11P	4K3Y	406	ARG
406	IBN	1A4G	373	ARG
406	SPN	3H72	721	ARG

### Loop Switching of Residues in Sialidase Active Sites

**Fig 3:**
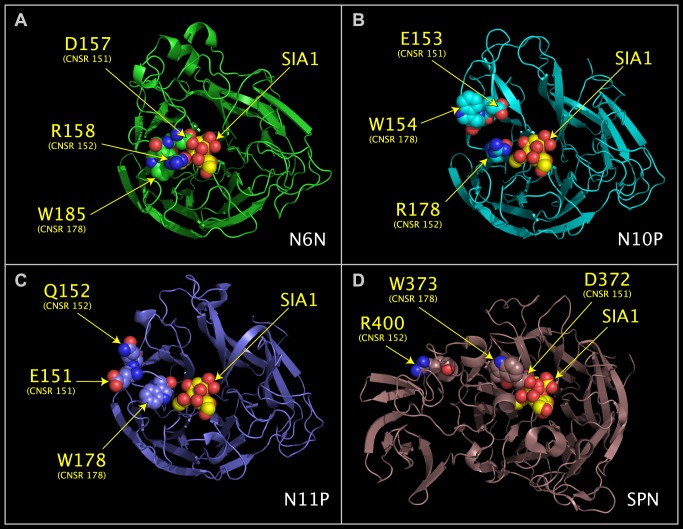
Loop swapping of CNSR151, CNSR152, and CNSR178. The structure ribbons of N6N (Fig. 3A), N10P (Fig. 3B), N11P (Fig. 4C), and SPN (Fig. 4D) are shown individually in their superposed positions. N6N residues D151, R152, and W178, shown in Fig. 4A, correspond to: N10P residues E153, R178, and W154, shown in Fig. 4B; N11P residues E151, Q152, and W178, shown in Fig. 4C; and SPN residues D372, R400 and W373, shown in Fig. 4D.

Residues D151 and R152 in the D151 loop have historically been considered to be a distinctive feature of influenza A neuraminidases. As shown in Fig. 3, and listed in Table 4, the positions of CNSR151, CNSR152, and CNSR178 residues in the protein chain can be different in functioning sialidases, such as N6N (Fig. 3A) and SPN (Fig. 3D). The SPN structure shows that there is structural precedent for the CNSR152 and CNSR178 having swapped positions in the chain of an active sialidase, i.e., a TRP is found at the chain position normally occupied by ARG, and an ARG is found at the chain position normally occupied by TRP. This switching of relative residue positions between proteins is signaled by negative offset values in Table 2. CNSR152 and CNSR178 residues in “swapped” positions in N10P (Fig. 3B) can reach the substrate in a geometry similar to residues in N6N and IBN. The positions of CNSR151, CNSR152, and CNSR178 in N6N, IBN, and SPN illustrate how functional groups on a single loop in one functional neuraminidase are found distributed to different loops in other, functional, neuraminidases. The replacement of an ARG residue by a GLN residue at CSNR152 in N11P (Fig. 3C) may be more significant than the swapping of CNSR152 and CNSR178 residues in the protein chain. Although N10P and N11P do not process MUNANA, their role as sialidases is uncertain as tests using exhaustive methods, such as the sialidase testing procedures suggested by Parker et al. [21], have not been reported.

**Fig 4:**
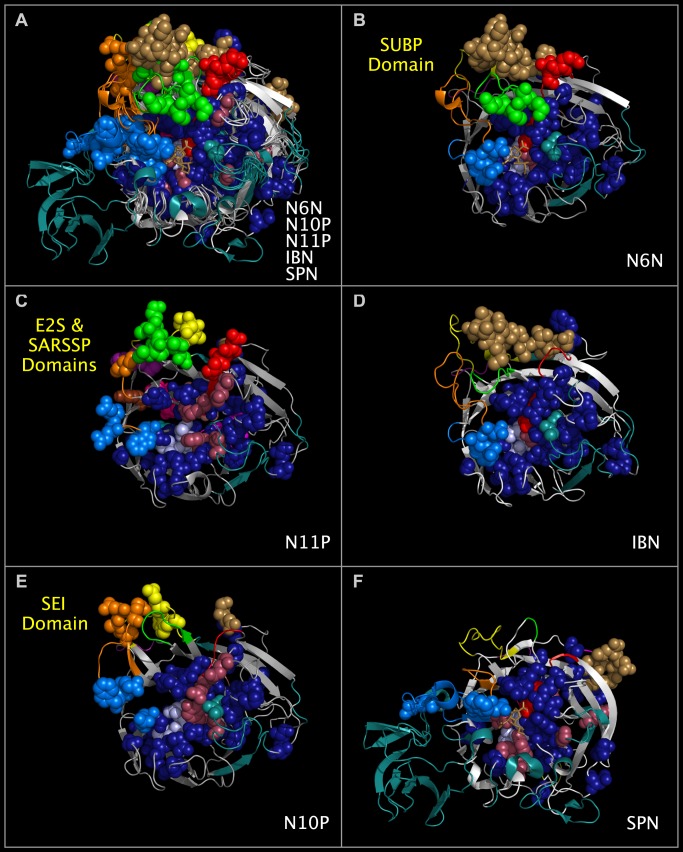
Consensus active site components and Upside VLR domains of N6N, N10P, N11P, IBN, and SPN. Panel 4A shows structure ribbons representing influenza A N10P [5], N11P [7], N6N [8], IBN [9]), and SPN [20] structures superposed using CNSA118:O, CNSA224:O, and CNSA276:O atoms. Atom spheres shown for each structure are identified by lowercase letters in Fig. 1. Structure ribbons and residue spheres of N10P, N11P, N6N, and IBN are color-coded as in the 'SPATIAL' row in Fig. 1. SPN residues are color coded as in the 'SPNSEQ' and 'SPN<>' rows in Fig. 1. Panel 4B shows substance P-like domains (green and red spheres) in the Upside VLR of N6N. Panel 4C shows E2S-like (green, brown, and orange spheres) and SARSSP-like (purple, red, and yellow spheres) in the Upside VLR of N11P. Panel 4D shows a C-terminal domain common to influenza B viruses (light brown colored spheres) in the Upside VLR of IBN. Panel 4E shows SEI-like domains (orange and yellow spheres) in the Upside VLR of N10P. Panel 4F shows a C-terminal domain common to bacterial viruses (light brown colored spheres) in the Upside VLR of SPN. Medium blue spheres adjacent to the consensus active site region in Panels 4A-4F are CNSR151, CNSR152, and CNSR 178 residues. Sialic acid sticks (colored medium brown) are shown in the consensus active site region of N6N, N10P, N11P, IBN, and SPN (Panel 4A), N6N (Panel 4B), and SPN (Panel 4F).

### Comparison of N6N, N10P, N11P, IBN, and SPN Structural Features

**Fig 5:**
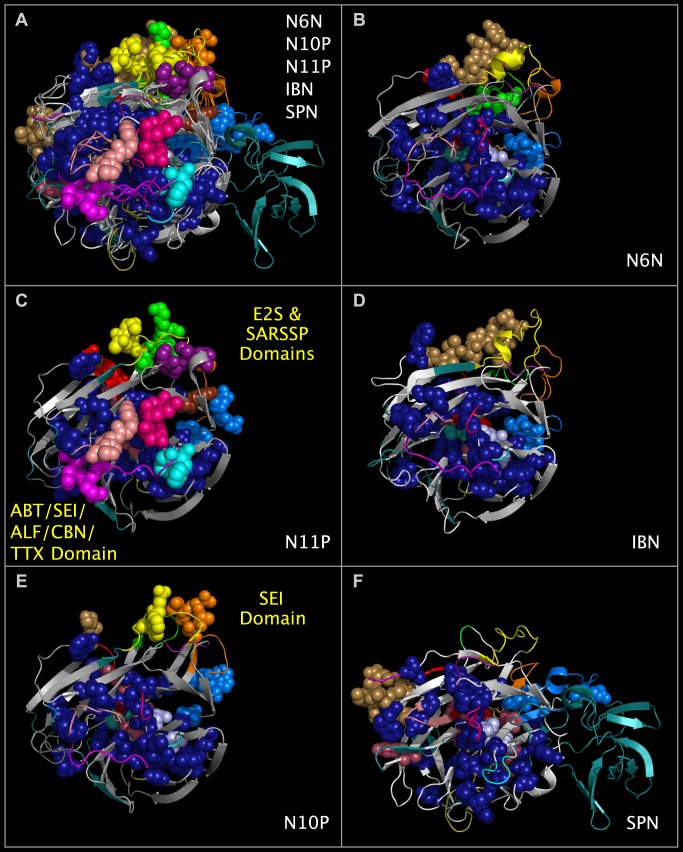
Downside VLR domains of N6N, N10P, N11P, IBN, and SPN. Panel 5A shows structure ribbons representing influenza A N10P [5], N11P [7], N6N [8], IBN [9]), and SPN [20] structures superposed using CNSA118:O, CNSA224:O, and CNSA276:O atoms. Atom spheres shown for each structure are identified by lowercase letters in Fig. 1. Structure ribbons and residue spheres of N10P, N11P, N6N, and IBN are color-coded as in the 'SPATIAL' row in Fig. 1. SPN residues are color coded as in the 'SPNSEQ' and 'SPN<>' rows in Fig. 1. Panel 5B shows the Downside VLR loops of N6N (colored magenta, hot pink, cyan, and salmon) in the foreground and the Upside VLR C-terminal light brown spheres also shown in Panel 4B in the background. Panel 5C shows residues found in ABT and SEI (colored magenta, hot pink, cyan, and salmon spheres) on the Downside VLR of N11P in the foreground and the Upside VLR E2S-like and SARSSP-like residues residues also shown in Panel 4C in the background. Panel 5D shows the Downside VLR loops of IBN (colored magenta, hot pink, cyan, and salmon) in the foreground and the Upside VLR C-terminal light brown spheres also shown in Panel 4D in the background. Panel 5E shows the Downside VLR loops of N10P (colored magenta, hot pink, cyan, and salmon) in the foreground and the Upside VLR SEI-like domain orange and yellow spheres also shown in Panel 4E at the top. Panel 5F shows the Downside VLR loops of SPN (colored magenta, hot pink, cyan, and salmon) in the foreground, an extra domain (colored teal) on the right hand side, and the Upside VLR C-terminal light brown spheres also shown in Panel 4F in the background.

Figs. 4 and 5 show the ribbon structures of N6N, N10P, N11P, IBN, and SPN monomers in a common reference orientation. The consensus active site relative positions, the regions of the proteins that contribute residues to the consensus active site area, and regions of the proteins that are not shared in N6N, IBN, SPN, N10P, and N11P can be seen by an examination of Figs. 1, 4, and 5. As can be seen from Figs. 4 and 5, the SPN structure has the same basic secondary structure and spatial position of the active site as N6N and IBN—but has several large structure extensions, colored deep teal and positioned in the sequence as shown in Fig. 1. The N6N, N10P, N11P, and IBN SIRs and corresponding SPN SIRs and SCRs, listed in Table 2 and shown in Figs. 4 and 5 as dark blue spheres, represent the invariant structural core of these related proteins. SPN has non-cysteine residues in the same conserved positions as the N6N, N10P, N11P, and IBN SIRs forming disulfide bridges; the SPN residues structurally corresponding to these cysteines are also colored dark blue. The corresponding CNSR151, CNSR152, and CNSR178 consensus active site residue spheres are shown in medium blue. The consensus invariant structural cores identified in N6N, N10P, N11P, IBN, and SPN are flanked by variable loop regions (“VLRs”) that are concentrated in two locations, which we refer to “Upside VLRs” and “Downside VLRs”. Fig. 4 shows the proteins with the “Upside” VLR presented. Fig. 5 shows the proteins with the “Downside” VLR presented. Light brown spheres, shown in Figs. 4A, 4B, 4D, 4F, 5A, 5B, 5D, and 5F, represent domains present on influenza A, influenza B, and bacterial neuraminidase structures; these domains are absent in N10P and N11P.

### Variable Loop Regions Contain Domains Found in Non-Neuraminidase Proteins

We found multiple, non-neuraminidase domains in the Upside VLRs: Staphylococcal Enterotoxin I (“SEI”) [22] in the N10P Upside VLR; hepatitis E2S protein (“E2S”) [23] and SARS spike protein [24] (“SARSSP”) in the N11P Upside VLR; and substance P (“SUBP”) [25] in the N6N Upside VLR. We also found toxin-like domains in N11P Downside VLRs; these toxin-like domains are present in alpha-bungarotoxin [26] (“ABT”), SEI [22], anthrax lethal factor [27] (“ALF”), clostridium botulinum neurotoxin [28] (“CBN”), and tetanus toxin [29] (“TTX”). Fig. 4 panel A shows the superposed structures of N6N, N10P, N11P, INB and SPN with Upside VLR residue spheres. Fig. 4 panel B shows the N6N Upside VLR residue spheres (colored red and green) with structural and sequence correspondences to SUBP. Fig. 4 panel C shows the N11P Upside VLR residue spheres (colored green, orange and brown) with structural and sequence correspondences to E2S. Fig. 4 panel C also shows the N11P Upside VLR residue spheres (colored yellow, red, and purple) with structural and sequence correspondences to SARSSP. Fig. 4 panel E shows the N10P Upside VLR residue spheres (colored yellow and orange) with structural and sequence correspondences to SEI.

Fig. 5 panel A shows the superposed structures of N6N, N10P, N11P, INB, and SPN with Downside VLR residue spheres from each structure. Fig. 5 panel C shows the N11P Downside VLR residue spheres (colored magenta, hot, pink, cyan and salmon) with structural and sequence correspondences common to ABT, SEI, ALF, CBN, and TTX. As can be seen by Fig. 5B, 5D, 5E, and 5F, this toxin-like domain is not found in N6N, N10P, IBN, or SPN.

### SEI Domain in N10P

**Fig 6:**
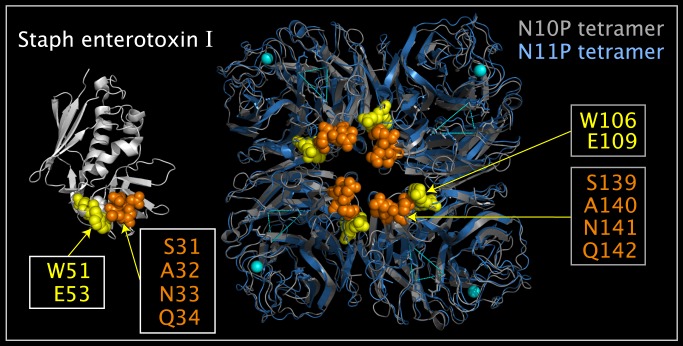
SEI domain and corresponding N10P tetramer Upside VLR residues with N11P tetramer reference. Shown are structure ribbons for SEI monomer (colored white), N10P tetramer (colored grey), and N11P tetramer (colored blue). The SEI monomer and N11P tetramer are unaltered crystal structures. The N10P tetramer was formed by translocating N10P monomers onto N11P monomers in the N11P tetramer. The yellow triangles on the N10P monomers are lines between CNSA118:O, CNSA224:O, and CNSA276:O whose superposition was used to orient N10P monomers into N11P tetramer positions. Residue spheres colored orange represent: SEI residues S31, A32, N33, and Q34; and corresponding N10P Upside VLR residues S139, A140, N141, and Q142. Residue spheres colored yellow represent: SEI residues W51and E53; and corresponding N10P Upside VLR residues W106 and E109.

**Fig 7:**
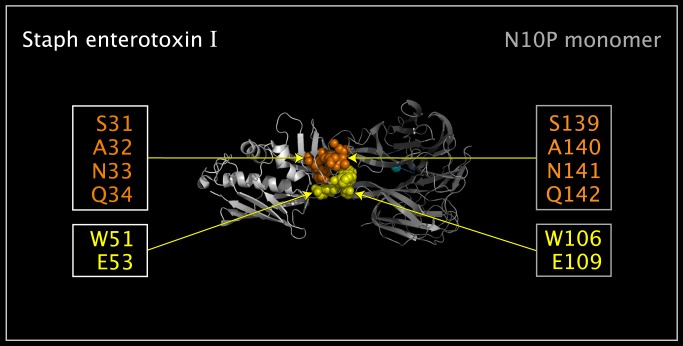
SEI and N10P Upside VLR residues in common spatial reference orientation. Shown are structure ribbons for superposed crystal structure SEI monomer (colored white) and N10P monomer (colored grey). Residue spheres colored orange represent: SEI residues S31, A32, N33, and Q34; and corresponding N10P Upside VLR residues S139, A140, N141, and Q142. Residue spheres colored yellow represent: SEI residues W51and E53; and corresponding N10P Upside VLR residues W106 and E109. The SEI monomer shown was superposed onto N11P tetramer Upside VLR residues using the atoms listed in Table 5.

Fig. 6 shows SEI, an N10 tetramer, and a N11 tetramer. In Fig. 6, the blue structure ribbon is the N11P tetramer (from 4K3Y.pdb [7]), and the grey tetramer is comprised of N10P monomers translocated onto the N11P tetramer by superposing the CNSA118:O, CNSA224:O, and CNSA276:O atoms, given in Table 1 and located at the vertices of the blue dotted-line triangles. Fig. 6 shows two sets of residues that comprise a domain that is common to N10P and SEI. In N10P, the SEI-like domain is highly structured and is distributed across two N10P monomers. For example, the N10P CB carbon atom of a A140 residue (spheres colored orange) fits into the six membered-ring of a W106 residue (spheres colored yellow) in an adjacent monomer. As shown in Fig. 7, the SEI domain can be mapped onto a corresponding N10P domain by mapping SEI atoms (W51:NE1, E52:CA, and Q34:O) onto N10P atoms (W106:NE1; E109: CA and S139:O). Atom numbers for the superposed atoms are given in Table 5. This superposition serves as a detailed example for the use of similar tables to be discussed. Fig. 7 shows the structural correspondence and orientation of the N10P and SEI proteins in a common reference orientation.

**Table 5:** Conserved atom geometry for SEI and N10P structures.

Atom Description	PDB	Chain	Res. #	Res. Type	Atom #	Atom Type
CNSA 106: NE1	4FVK	A	106	TRP	207	NE1
	2G9H	D	51	TRP	3509	NE1
CNSA 109: CA	4FVK	A	109	GLU	225	CA
	2G9H	D	53	GLU	3524	CA
CNSA 139: O	4FVK	A	139	SER	465	O
	2G9H	D	34	GLN	3373	O

### E2S and SARSSP Domains in N11P

**Fig 8:**
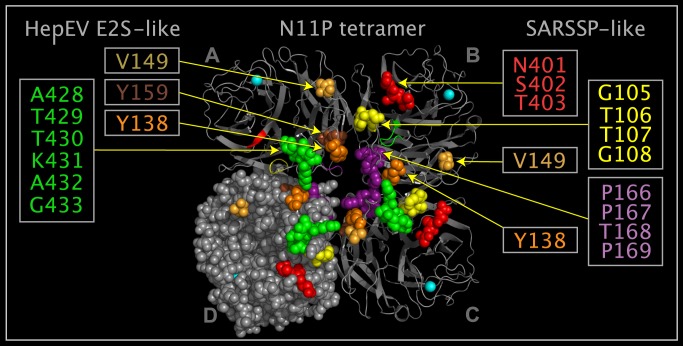
E2S domains, SARSSP domains, and corresponding N11P Upside VLR residues. Shown are N11P tetramer crystal structure ribbons colored grey, associated calcium atoms are colored cyan, and spheres depicting selected N11P Upside VLR residues colored grey with the following exceptions: in monomers in positions “A”, ”B”, ”C”, and ”D”, Y138 is colored orange, V149 is colored light tangerine and calcium atoms are colored cyan; in monomers in positions “A”, ”C”, and ”D”, ALA428-G433 spheres are colored green, and Y159 is colored brown; and in monomers in positions ”B”, ”C”, and ”D”, G105-G108 are colored yellow, P166-P169 are colored purple, and N401-T403 are colored red. Residues between Y138 and V149 are missing in the crystal structure in monomers in positions “A”, ”B”, and ”D”, and the structure is disjoint. In monomer in position “C”, residue G139 is between residues Y138 and V149 and the crystal structure of this monomer chain is presented as contiguous. Green, brown, and orange spheres correspond to an E2S-like domain. Yellow, purple, and red spheres correspond to a SARS spike protein-like domain.

**Fig 9:**
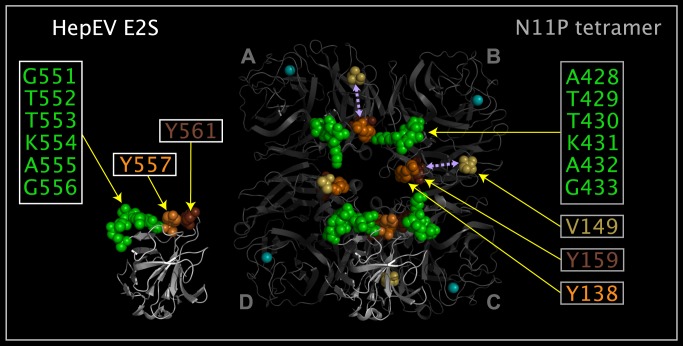
E2S and N11P Upside VLR residues in common spatial reference orientation. Shown are two E2S monomers (structure ribbons colored white) and a N11P tetramer (structure ribbons colored grey and associated calcium atoms colored cyan) from crystal structures. One E2S monomer is shown apart from the N11P tetramer and the other E2S monomer is shown with E2S residues superposed onto N11P tetramer Upside VLR residues using the atoms listed in Table 6. N11P Upside VLR residue spheres depict: Y138 colored orange, V149 colored light tangerine, Y158 colored brown, and ALA428-G433 spheres colored green. E2S residue spheres in the stand-alone and superposed monomers depict: Y557 colored orange, Y561 colored brown, and G551-G556 colored green. V149 light tangerine residue spheres in N11P monomers in positions “A”, ”B”, ”C”, and ”D” and arrows in monomers in positions “A” and ”B” are shown as a reference to residues missing between Y138 and V149 in the N11P monomers and have no structural correspondence in E2S. The E2S monomer residues spheres shown are superposed onto N11P residue spheres from two N11P monomers.

**Fig 10:**
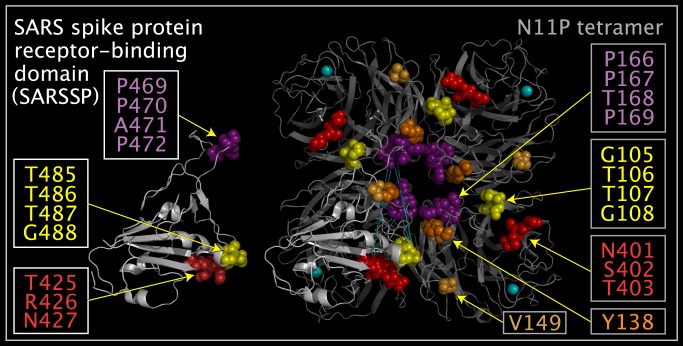
SARSSP and N11P Upside VLR residues in common spatial reference orientation. Shown are two SARSSP monomers (structure ribbons colored white) and a N11P tetramer (structure ribbons colored grey and associated calcium atoms colored cyan) from crystal structures. One SARSSP monomer is shown apart from the N11P tetramer and the other SARSSP monomer is shown with SARSSP residues superposed onto N11P tetramer Upside VLR residues using the atoms listed in Table 7. N11P Upside VLR residue spheres depict: Y138 colored orange, V149 colored light tangerine, G105-G108 colored yellow, P166-P169 colored purple, and N401-T403 colored red. SARSSP residue spheres in the stand-alone and superposed monomers depict: T485-G488 colored yellow, P469-P472 colored purple, and T425-N427 colored red. Y138 orange and V149 light tangerine residue spheres in N11P are shown as a reference to residues missing between Y138 and V149 in the N11P monomers and have no correspondence in the SARSSP monomer.

N11P Upside VLR residues correspond to residues in the reported structures of E2S [23] and SARS spike protein [24]. The E2S domain is formed from three sets of residues (A428-G433, Y138, and Y159). Movement of a N11P loop containing six E2S-like domain residues (A428-G433) exposes SARSSP-like domain residues (G105-G108, P166-P169, and N401-T403). Fig. 8 shows the spatial relationship between the E2S-like and SARSSP-like domains. Fig. 9 shows E2S and corresponding N11P Upside VLR residues presented in different and common reference orientations. The common reference orientation of E2S and N11P residues is achieved by superposing the atoms with common distributed geometry listed in Table 6. Fig. 10 shows SARSSP and corresponding N11P residues presented in different and common reference orientations. The common reference orientation of SARSSP and N11P residues is achieved by superposing the atoms with common distributed geometry that are listed in Table 7. The loops containing residues P105-P108 in N11P and residues P469-P472 in the SARSSP are mobile. The P469-P472 residues in SARSSP could easily reposition to bind within a monomer, instead of across monomers as shown in Fig. 10.

**Table 6:** Conserved atom geometry for Hep E E2S and N11P structures.

Atom Description	PDB	Chain	Res. #	Res. Type	Atom #	Atom Type
CNSA 138: CB	4K3Y	A	138	TYR	439	CB
	3RKD	A	557	TYR	754	CB
CNSA 431: CA	4K3Y	C	431	LYS	8001	CA
	3RKD	A	554	LYS	733	CA
CNSA 430: CB	4K3Y	C	430	THR	7997	CB
	3RKD	A	553	THR	729	CB

**Table 7:** Conserved atom geometry for SARSSP and N11P structures.

Atom Description	PDB	Chain	Res. #	Res. Type	Atom #	Atom Type
CNSA 107: O	4K3Y	C	107	THR	5637	O
	3SCK	F	486	THR	12385	O
CNSA 403: O	4K3Y	C	403	THR	7764	O
	3SCK	F	425	THR	11880	O
CNSA 167: N	4K3Y	D	167	PRO	8738	N
	3SCK	F	470	PRO	12247	N

### Toxin Domains in N11P

**Fig 11:**
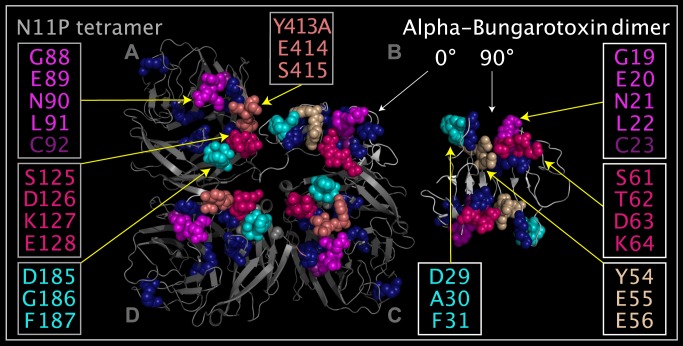
Corresponding residues in ABT dimer and N11P Downside VLR. Shown are two ABT dimers (structure ribbons colored white with dark blue spheres representing disulfide bridges) and a N11P tetramer (structure ribbons colored grey and with dark blue spheres representing disulfide bridges) from the crystal structures. One ABT dimer is shown apart from the N11P tetramer and the other ABT dimer is shown at an angle that is 90 degrees rotated from the first dimer in position “B”, i.e. positioned with the ABT dimer superposed onto and substituting for the N11P monomer in position “B” in the tetramer. Atoms used to superpose the ABT dimer into the position that would be occupied by the N11P “B” monomer are listed in Table 8. N11P Downside VLR residue spheres in monomer positions “A”, “C”, and “D” depicting: G88-L91 colored magenta, C92 colored plum, S125-E128 colored hot pink, D185-F187 colored cyan, and Y413A-S415 colored salmon. Corresponding ABT residue spheres in the two dimers depicting: G19-L22 colored magenta, C23 colored plum, S61-K64 colored hot pink, D29-F31 colored cyan, and Y54-E56 colored tan. N11P monomers in the “A” and “D” positions are shown in the crystal structure positions. In the N11P monomer in the “C” position, the Y413A-S415 residues (colored salmon) have been rotated into the same position relative to G19-L22 (colored magenta) and D29-F31 (colored cyan) as in corresponding residues of ABT crystal structure dimer (shown in the “B” position) in order to illustrate that small movements of the mobile N11P Downside VLR residues can produce nearly identical relative residue presentation to ABT.

**Fig 12:**
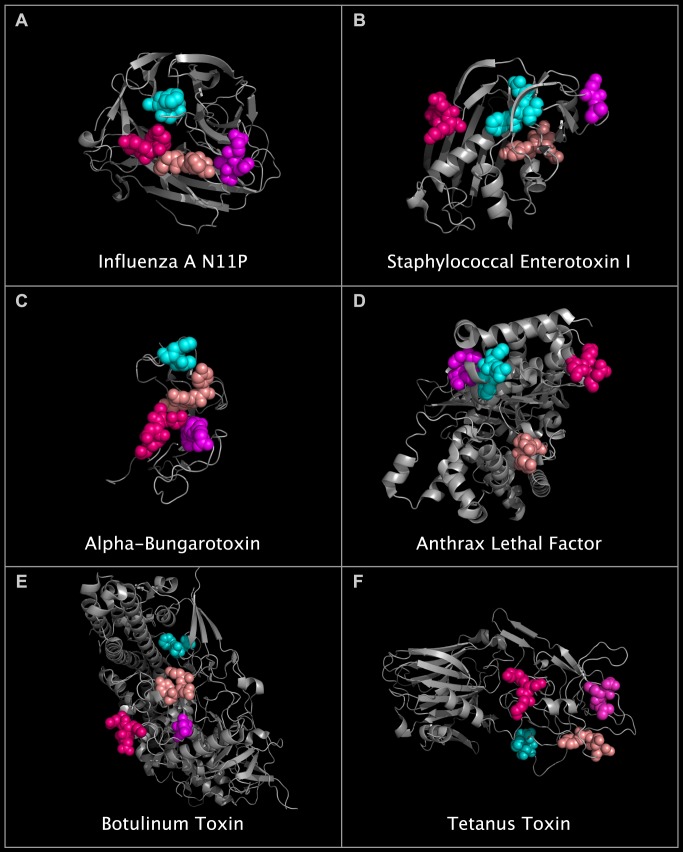
N11P Downside VLR residues and corresponding SEI, ABT, ALF, CBN, and TTX residues. Shown are grey structure ribbons depicting: N11P monomer (panel A), SEI monomer (panel B), ABT dimer (panel C), ALF monomer (panel D), CBN monomer (panel E), and TTX monomer (panel F). Corresponding residue spheres in each panel are identified and colored according to Table 9.

N11P Downside VLR residues and residues in alpha-bungarotoxin dimers have common local spatial occupancy of residues as shown in Fig. 11. Fig. 11 shows three monomers of the N11P tetramer in positions A, C, and D. In place of the N11P monomer in the “B” position is a dimer of alpha-bungarotoxin superposed onto the N11P monomer, not displayed, in the “B” position. This alpha-bungarotoxin dimer was superposed onto the N11P monomer in the “B” position using the atoms listed in Table 8. The N11P monomer in the “C” position shows the N11P residues Y413A-S415 moved to a position relative to N11P residues D85-F87 that is the same as the relative position between ABT residues Y54-E56 and ABT residues D29-F31. As the residues in the N11P Downside VLR are flexible, the spatial relationship between the groups of residues is not fixed. As can be seen from Fig. 11, there is a strong structural correspondence between the individual N11P domains mapped onto ABT, suggesting that movement of the mobile loops produces the same combined domain structure in N11P and ABT. This set of residues is present in other toxins suggesting its importance. Table 9 lists residue correspondences between N11P, SEI, ABT, ALF, CBN, and TTX. Fig. 12 shows that these structurally characterized toxins present similar clusters of N11P Downside VLR residues on mobile loops.

**Table 8:** Conserved atom geometry for alpha-bungarotoxin and N11P structures.

Atom Description	PDB	Chain	Res. #	Res. Type	Atom #	Atom Type
CNSA 30: O	4K3Y	D	185	ASP	8885	O
	2ABX	A	30	ASP	222	O
CNSA 55:OE(1,2)	4K3Y	D	414	GLU	10585	OE1
	2ABX	A	55	GLU	407	OE2
CNSA 20: CA	4K3Y	D	89	GLU	8224	CA
	2ABX	B	20	GLU	681	CA

**Table 9:** Toxin and lethal factor domains found in N11P.

Domain	Fig. 6 Panel	PDB	Chain	Residue		Residue		Residue		Residue		Residue	
Domain I (cyan)	A	4K3Y	A	(G)		E	89	N	90	L	91		
	B	2G9H	D	(D)		(K)		N	26	L	27		
	C	2ABX	A	(G)		E	20	N	21	L	22		
	D	1YQY	A	(G)		N	722	N	723	L	724		
	E	3ZUQ	A	(I)		(K)		N	385	L	386		
	F	1DLL	A	(F)		N	1219	N	1200	L	1221		
Domain II (salmon)	A	4K3Y	A	Y	414	E	415	S	416				
	B	2G9H	D	Y	195	E	196	D	197				
	C	2ABX	B	Y	54	E	196	E	197				
	D	1YQY	A	Y	650	E	651	Q	652				
	E	3ZUQ	A	Y	421	E	422	E	423				
	F	1DLL	A	Y	1258	D	1259	D	1260				
Domain III(deep pink)	A	4K3Y	A	(C)		S	125	D	126	K	127	E	128
	B	2G9H	D	S	109	T	110	D	111	K	112	(I)	
	C	2ABX	A	S	61	T	62	D	63	K	64	(C)	
	D	1YQY	A	(L)		(L)		D	701	K	702	N	703
	E	3ZUQ	A	(I)		S	401	D	402	K	403	D	404
	F	1DLL	A	(L)		K	1295	D	1296	K	1297	(I)	
Domain IV(magenta)	A	4K3Y	A	D	185	G	186	F	187				
	B	2G9H	D	D	63	I	64	F	65				
	C	2ABX	B	D	30	A	31	F	32				
	D	1YQY	A	D	716	I	718	F	719				
	E	3ZUQ	A	D	488	I	489	F	490				
	F	1DLL	A	D	1187	S	1188	F	1189				

### Substance P Domain in N6N

**Fig 13:**
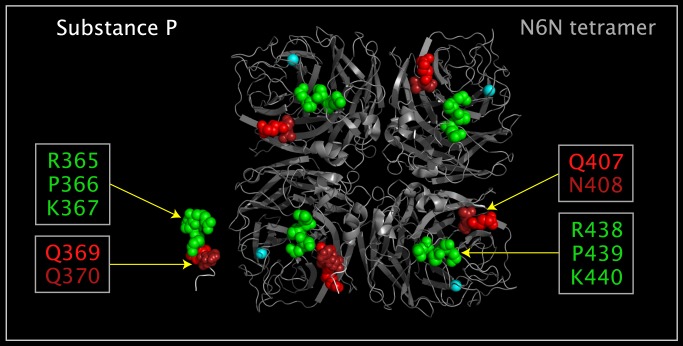
Reoriented Substance P residues and corresponding N6N Upside VLR residues. Shown are a crystal structure N6N tetramer (structure ribbons colored grey), a crystal structure substance P apart from the N6N tetramer, and a model-built substance P monomer superposed on a monomer in the N6N tetramer. The model built structure coordinates are given as a PDB file, “WSUBP.pdb”, available as supporting information **S2 File**. The atoms used to map WSUBP.pdb onto the N6N monomer are given in Table 10. The model building consisted of reorienting the first 3 residues of substance P, R365-K367 (colored green), to exactly match the corresponding N6N residues, R438-K440 (colored green), and leaving the rest of the structure as in the crystal structure. N11P residues, Q 407 and N408 (colored red), correspond to substance P residues, Q369 and Q370 (colored red).

Domains similar to those found in the substance P structure [25] were found in the Upside VLR of N6N. Fig. 13 shows substance P and the presentation of a substance P-like domain in N6N. The small (11 amino acids) substance P is highly flexible and multiple N-terminal conformers can occupy the same volume. In order to map substance P domains to N6N domains, substance P residues R365, P366, and K367 (the first, second, and third residues of substance P) have been reoriented to place the substance P atoms in the same configuration as the corresponding residues (R438, P439, and K440) in N6N. All other residues and all main chain atoms in both structures are in the same relative positions as in the the crystal structures. The coordinates for this model-built and translocated structure were output as a pdb file, “WSUBP.pdb”, available as supporting information **S2 File**. Fig. 13 shows the overlap of the R365, P366, K367, Q369, and Q370 residue spheres of WSUBP.pdb with the corresponding residue spheres of N6N (R438, P439, and K440, Q407, and N408) after superposition using the atoms provided in Table 10.

**Table 10:** Conserved atom geometry for Substance P and N6N structures.

Atom Description	PDB	Chain	Res. #	Res. Type	Atom #	Atom Type
CNSA 2438: CA	1W1X	C	2438	ARG	8715	CA
	WSUBP	B	365	ARG	5869	CA
CNSA 2439: CA	1W1X	C	2439	PRO	8726	CA
	WSUBP	B	366	PRO	5895	CA
CNSA 2440: CA	1W1X	C	2440	LYS	8733	CA
	WSUBP	B	367	LYS	5909	CA

## Conclusions

Using common spatial occupancy of distributed and localized sets of atoms, divergent structures can be aligned and putative functional domains can be identified. Atoms with common distributed spatial occupancy can be used to superpose structures. Once structures are superposed in a common orientation, structural variation within the superposed structures can be identified and interpreted. Superposition of conserved atoms in related structures allows small molecules and atoms complexed with one structure to be mapped to another structure. Superposed bound molecules may suggest putative binding sites in functionally uncharacterized proteins. Once structures are superposed, structural invariants can be identified and used as markers for the alignment of non-homologous structures or sequences. In this way, structures or sequences that have little or no sequence similarity can be aligned. The identification of structural invariants allows the identification of any highly divergent areas of the protein. Proteins outside of a functionally related group may be used to interpret the function of divergent structures.

Proteins within a class, such as viral neuraminidases, can be interpreted using structures outside of the class such as bacterial neuraminidases. This is a particularly important capability for the assessment of functional changes in emerging viruses. N10P and N11P structural reports could not identify an active site, functioning or disabled, without superposition of the structures. The bacterial neuraminidase structure, when aligned with influenza A and B neuraminidases, provides important information on the natural structural variation in active sites of functioning sialidases. Alternative configurations of residues performing the same function must be considered when evaluating proteins from emergent pathogens or divergent proteins within a class. Changes in geometry of an active site may impact substrate preference and must be considered when assessing divergent enzyme function by substrate processing, especially by artificial substrate processing.

Structural analysis using common spatial occupancy can identify structural features in proteins that may impact function. It is not clear whether N10P or N11P are functional or dysfunctional neuraminidases as this has not been adequately tested and reported. N10P may not be able to bind and process MUNANA as the large size and stereochemistry of MUNANA may prevent the closure of the loop containing the CNSR178 residue (N10P residue W154). Changes in presentation of the CNSR152 and CNSR178 residues would be expected to impact the ability of MUNANA to bind to a neuraminidase. In addition, N10P and N11P display structural features that suggest that, if they are even active, their substrate preference may be altered relative to other viral sialidases as key residue presentation to the active site has features found in bacterial sialidases (e.g. SPN). MUNANA is an artificial substrate consisting of a proto-fluorophore linked to Neu5Ac. S. *pneumoniae* produces three sialidases Nan A, Nan B, and Nan C with different substrate requirements. Nan A is promiscuous (accepting many sialosides), Nan B prefers alpha 2,3 sialosides, and NanC must process a substrate and hydrate it to form Neu5Ac before cleaving it. The Neu5Gc form of sialic acid is produced by many non-human mammals instead of the Neu5Ac (the most common sialic acid in humans) [21]. Alternatives to MUNANA as a substrate as an assay for N10P and N11P must be considered in light of the variation of presentation of active site components on alternate loops in their respective binding pockets.

Regardless of activity, or lack thereof, N10P and N11P have CNS R292T mutations that are expected to confer resistance to oseltamivir, zanamivir, and peramivir. If N10P-like and N11P-like proteins are present in infections with mixed virus populations, failure to identify functioning, drug-resistant viruses could facilitate spread of a resistant virus to health care workers that have prophylactically taken currently available antivirals with the mistaken belief that they are protected. In mixed virus populations, there is the possibility that H17N10 and H18N11 viruses can reassort and rescue drug sensitive viruses or be rescued by resistant bacterial neuraminidases. Resistant bacterial neuraminidases have been found to rescue sensitive influenza viruses from inhibition by the neuraminidase inhibitor, zanamivir [3]. H17N10 and H18N11 viruses should be carefully monitored for this reason. It remains to be determined whether N10P and N11P sites containing active site components are functionally active neuraminidase sites or represent vestigial active sites made obsolete by the incorporation of new cell entry domains.

Our identification of groups of localized residues in N10P and N11P having conserved spatial occupancy with non-influenza protein residues allowed the identification of putative cell entry domains in the N10P and N11P structures. These cell entry domains may be strategic in N10P and N11P in the absence of, change in, or reduction in sialidase activity. Loss of N10P and N11P sialidase activity coupled to the appearance of cell entry domains from bacterial toxins and other viruses is unprecedented.

The strong structural correspondence of SEI domains and N10P domains, even without altering the crystal structure positions of the residues in the corresponding domains to maximize overlap, suggest that H17N10 influenza virus may enter cells by binding to human MHC class II molecules in a manner similar to that of SEI. The SEI proteins bind to human MHC class II proteins and they were co-crystallized with the MHC class II proteins in the crystal structure [22].

The strong structural correspondence of E2S, SARSSP, and toxin-like domains and N11P domains, even without altering the crystal structure positions of the residues in the corresponding domains to maximize overlap, suggest that H18N11 influenza virus may enter cells by binding to an expanded set of human cellular receptors, including ACE2 and acetylcholine receptors. The identification of the similar residue domains in SEI, ABT, ALF, CBN, and TTX suggests that these domains are important, conserved structures in these toxins. The fact that multiple toxins have similar domains to N11P domains suggests that the H18N11 influenza virus may, at the least, have the structural components necessary to enter cells via acetylcholine receptors. Whether these domains on multiple mobile loops enable viruses containing them to enter cells via the acetylcholine receptor should be investigated.

The strong structural correspondence of substance P residues and N6N residues, after altering the crystal structure positions of three of eleven of the highly flexible substance P residue side chains, suggest that N6N may have the ability to enter cells by binding to tachykinin receptors. The presentation of binding components that can bind simultaneously, as might occur when substance P-like domains are presented by an N6N tetramer, may cause a dramatic increase in binding affinity even if the number of residues in the individual binding domain is small. Multiple small binding domains, presented on an influenza virus, in a geometry where they can bind to more than one receptor simultaneously, would have an overall dramatically increased affinity. If n is the binding affinity of one domain, two domains binding simultaneously and cooperatively would be expected to produce approximately (n^2^—n) binding affinity. For this reason, clusters of atoms that can achieve a similar common spatial occupancy are significant even if the cluster is formed from atoms from small numbers of residues on different loops.

The non-influenza virus-like domains that we have identified in N10P and N11P are important to consider in developing diagnostic antibodies and therapeutic vaccines. The presence of these domains suggests that proteolytically released N11P may possibly be detected by anti-ABT and other toxin-related antibodies.

This method of relating distributed and local common spatial occupancy is general and can be applied to any set of structures, regardless of the size, complexity, particular orientation, distribution, or diversity of local structure of the components. The use of atom sets to superpose structures based on similar relative geometry of atoms is a powerful tool. The method of identifying the common and divergent spatial occupancy of atoms and proteins provides rapid, effective assessment of divergent emerging virus features and provides testable hypotheses about how viral sequence changes are related to viral trait changes such as cellular receptor preference. The method, demonstrated by the analysis of N10P and N11P, is a general method for evaluating proteins.

## Materials and Methods

### Overview of Method: Common Spatial Occupancy

Common spatial occupancy of atoms in structures consists of sets of atoms from structures having conserved distribution in space. The atoms with the same relative positions can be distributed or localized in one structure (e.g. in a monomer) or between multiple structures (e.g. across a dimer or other intermolecular association). We identified atoms with common spatial distribution and then used these atoms to orient structures (N6N, N10P, N11P, IBN, and SPN) relative to one another. These aligned structures were used to align sequences with no structures (e.g., other Influenza A sequences as shown in Fig. 1) and to identify and characterize areas of structural similarity and deviation. Common spatial occupancy was further used to identify structural correlates of structural deviation.

### Method for Determination of Common Distributed Relative Spatial Occupancy

**Fig 14:**
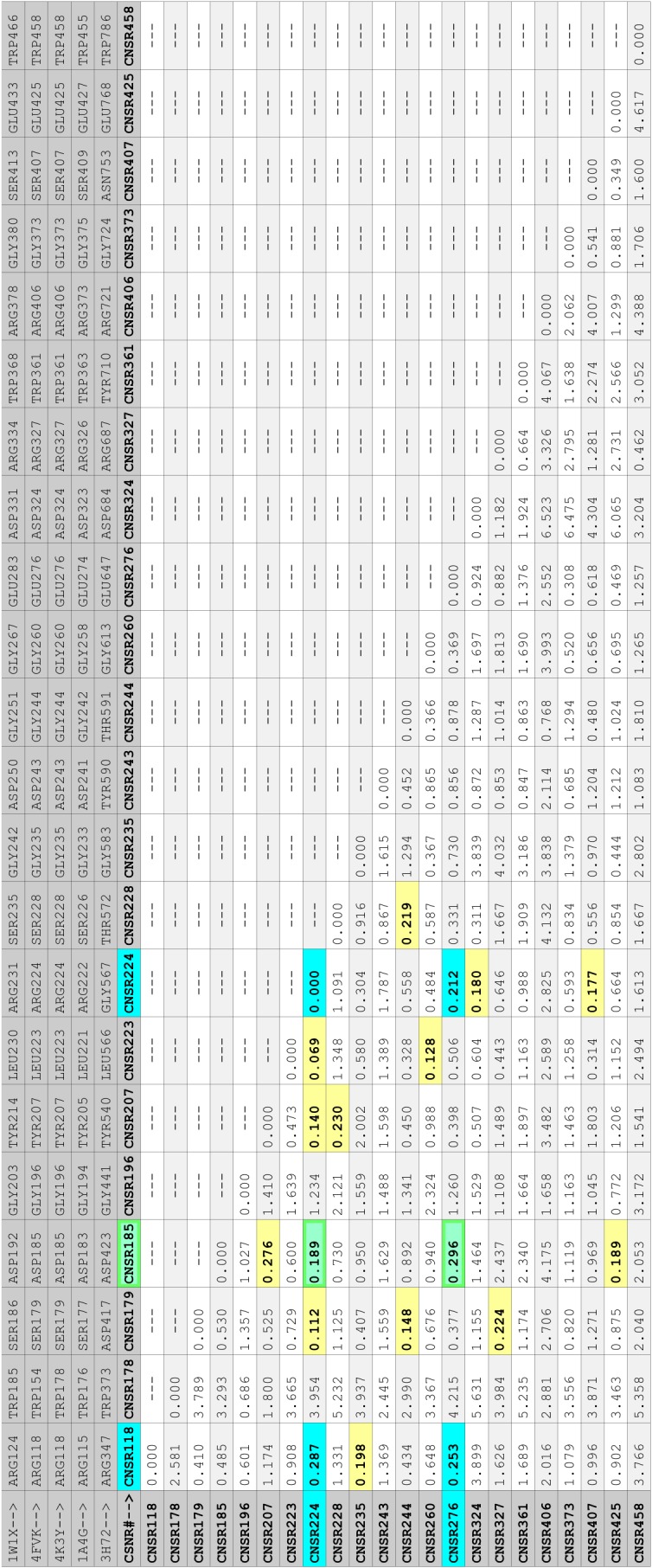
Interatomic distance population standard deviations for selected N6N, N10P, N11P, IBN, and SPN atoms. Shown are the standard deviations of the interatomic distances for specific main chain oxygens of N6N, N10P, N11P, IBN, and SPN residues; these oxygens were selected if the Table 2 row for their associated residue does not contain cysteines, prolines, or missing residues. The standard deviations colored in cyan correspond to the minimal standard deviation of the distances between the CNSA118:O, CNSA224:O, and CNSA276:O atoms. The standard deviations colored in green correspond to the minimal standard deviation of the distances between the CNSA185:O atom and each of the CNSA224:O and CNSA276:O atoms. The CNSA118:O, CNSA224:O, CNSA276:O, and CNSA185:O atoms form a tetrahedron. Values under 3.00 are colored and, unless colored cyan or green as above, default to yellow.

**Fig 15:**
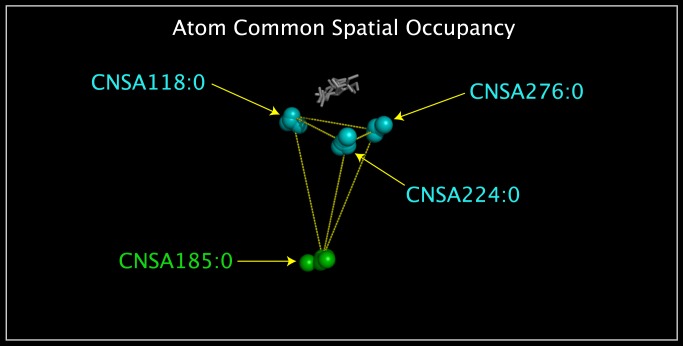
Overlapping CNSA118:O, CNSA224:O, CNSA276:O, and CNSA185:O atom tetrahedrons. Shown are the superposed CNSA118:O, CNSA224:O, CNSA276:O, and CNSA185:O atoms from N6N, N10P, N11P, IBN, and SPN. The CNSA118:O, CNSA224:O, and CNSA276:O atoms are colored cyan. The CNSA185:O atoms are colored green. The CNSA118:O, CNSA224:O, CNSA276:O, and CNSA185:O atoms form a tetrahedron. Also shown are yellow lines drawn between the N6N CNSA118:O, CNSA224:O, CNSA276:O, and CNSA185:O atoms. The combined superposed substrates and inhibitors from the superposed structures are shown as grey sticks.

**Fig 16:**
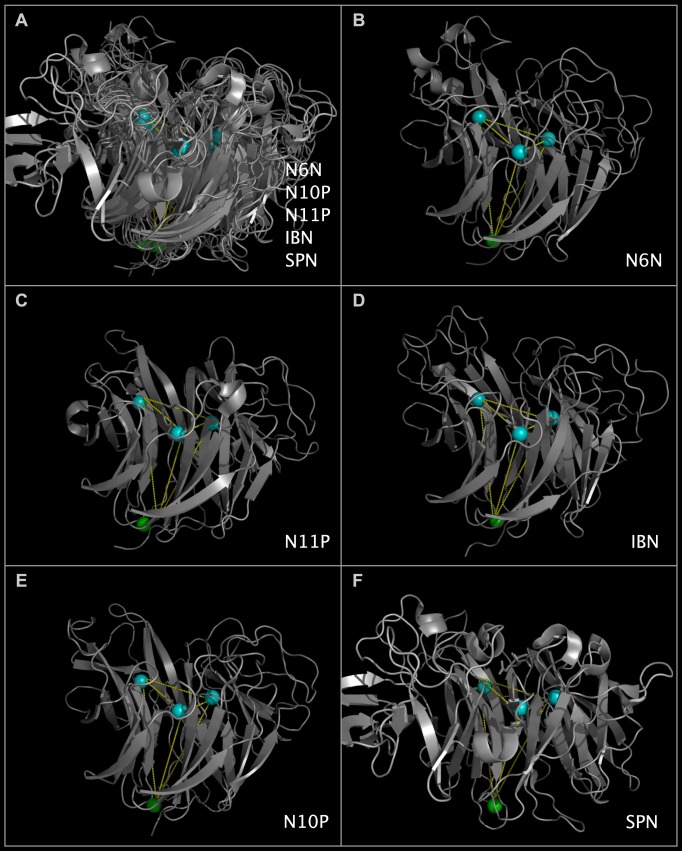
CNSA118:O, CNSA224:O, CNSA276:O, and CNSA185:O atom tetrahedrons in N6N, N10P, N11P, IBN, and SPN. Panel A shows the superposed N6N, N10P, N11P, IBN, and SPN structure ribbons colored grey, the CNSA118:O, CNSA224:O, and CNSA276:O atoms colored cyan, the CNSA185:O atoms colored green, and the lines between N6N CNSA118:O, CNSA224:O, CNSA276:O, and CNSA185:O atoms colored yellow. Panels B-E show individual N6N, N10P, N11P, IBN, and SPN structures. In Panels B-E, the structure ribbons are colored grey, the corresponding CNSA118:O, CNSA224:O, and CNSA276:O atoms are colored cyan, the corresponding CNSA185:O atoms are colored green, and actual or superposed lines between N6N CNSA118:O, CNSA224:O, CNSA276:O, and CNSA185:O atoms are colored yellow (forming a tetrahedron).

**Fig 17:**
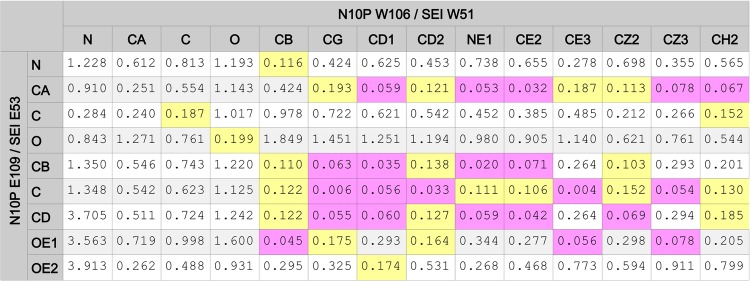
Interatomic distance population standard deviations for selected N10P and SEI atoms. Shown are the standard deviations of the interatomic distances for N10P residue atoms in W106 and E109 and the interatomic distances SEI residue atoms in W51 and E53. The standard deviations shown in magenta correspond to the minimal standard deviations less than 0.1. The standard deviations shown in yellow correspond to the minimal standard deviations less than 0.2. The TRP and GLU residues in the N10P and SEI proteins are in the same relative spatial orientation indicated by a large cluster of low interatomic distance standard deviations despite the fact that these residues are separated by two amino acids in N10P and one amino acid in SEI.

The N6N, N11P, N11P, IBN, and SPN structures were reoriented relative to one another by superposition of atoms with common distributed relative spatial occupancy in these structures. The CNSR118:O, CNSR224:O, and CNSR276:O atoms, listed in Table 1, can be identified by calculating the distances between all atom pairs in each structure and then identifying sets of spatially distributed (not the same or contiguous) atoms with identical or near-identical spatial distribution. Fig. 14 shows the standard deviation of the interatomic distances between the main chain oxygen atoms of specific residues in Table 2 that are found to be conserved among N6N, N10P, N11P, IBN, and the corresponding atoms in SPN; these residues were selected to be from rows in Table 2 that do not contain cysteines, prolines, or missing residues. **S3 File** lists, in the order of data calculation, these selected main chain oxygen atoms, the interatomic distances between these oxygen atoms, and the standard deviation values of the set of these interatomic distances. Fig. 14 highlights low standard deviation values (i.e. values under 0.300) in color: yellow by default and cyan and green for standard deviation values of distances between pairs of CNSA118:O, CNSA224:O, CNSA276:O, and CNSA185:O atoms. Fig. 15 shows the spatial presentation of atom pairs that correspond to the cyan and green low standard deviation values in Fig. 14. These atoms form a tetrahedron. Superposition of the corresponding CNSA118:O, CNSA224:O, and CNSA276:O atoms, listed in Table 1, is sufficient to place the N6N, N10P, N11P, IBN, and SPN structures into a common reference orientation; the use of the CNSA185:O atom does not improve the result. Fig. 16 shows the N6N, N10P, N11P, IBN, and SPN structures oriented into a common spatial reference orientation using their corresponding CNSA118:O, CNSA224:O, and CNSA276:O atoms. Fig. 16 also shows the CNSA185:O atom and the tetrahedron formed by the CNSA118:O, CNSA224:O, CNSA276:O, and CNSA185:O atoms for reference. Figs. 14–17 illustrate a method used to select atoms with common spatial distribution that can be used to superpose sequence related and unrelated structures.

### Method for Determination of Common Localized Relative Spatial Occupancy

N10P, N11P, and N6N structures were placed into a common reference orientation with non-influenza protein binding domains by superposition of atoms with common localized relative spatial occupancy. Local atomic correspondences can be identified independently from, and without use of, the chain position of corresponding residues. A detailed example of this method is given for tryptophan and glutamic acid residues having different relative chain positions in N10P and SEI. Fig. 17 shows the standard deviation of N10P and SEI sets of the distances between TRP and GLU residues. The clustering of low standard deviation values indicates that these residues are in the same spatial position relative to one another in both structures -even though the residues are separated by two residues in N10P and one residue in SEI. **S4 File** contains excerpted atoms, interatomic distances, and standard deviation values presented in Fig. 17. This method shows that it is the relative spatial positioning of atoms in residues rather than the chain positions of the residues that determines spatial structural correspondence. This use of common spatial occupancy between clustered atoms can be used to identify similar distributions of atoms, regardless of whether the atoms are contained in one monomer or distributed across any combination of molecules. Sets of atoms with localized common spatial occupancy are not restricted to the same molecule and can be distributed among associated molecules. Examples of localized intermolecular contacts identified are: N11P monomer and ABT dimer atoms presented in Fig. 11 and Table 8; and N11P dimer and SARSSP monomer atoms presented in Fig. 10 and Table 6. Substrate-protein atom contacts with sialic acid in the N6N-sialic acid complex in the superposed structures can also be evaluated using this method.

Molecular display programs can also be used to superpose molecules. Structural alignment and overlap can also be confirmed visually or by using standard deviation of atom pairs.

## Supporting Information

S1 File Figure Abbreviations, References, Sequence Identifiers, and Sequence Descriptions. List of sequences used, with sources and distribution of sequence groups in Fig. 1.
(PDF)

S2 File “WSUBP.pdb”. Coordinates, in pdb format, of 11 residues of substance P with reoriented R1, P2, and K3 side chains.
(PDB)

S3 File N6N, N10P, N11P, IBN, and SPN Example of Common Distributed Relative Spatial Occupancy. List of selected N6N, N10P, N11P, IBN, and SPN main chain oxygen atoms, interatomic distances, and interatomic distance population standard deviation values, as seen in Figs. 14–16.
(TXT)

S4 File N10P and SEI Example of Common Localized Relative Spatial Occupancy. List of atoms in specific TRP and GLU residues in N10P and SEI, interatomic distances, and population standard deviation values, as seen in Fig. 17.
(TXT)
