# Supplementary material for: Using Common Spatial Distributions of Atoms to Relate Functionally Divergent Influenza Virus N10 and N11 Protein Structures to Functionally Characterized Neuraminidase Structures, Toxin Cell Entry Domains, and Non-Influenza Virus Cell Entry Domains
Source: PLoS One. 2015 Feb 23;10(2):e0117499. doi: 10.1371/journal.pone.0117499 (PMC4337911; doi:10.1371/journal.pone.0117499)
Supplement: S1 File — List of sequences used, with sources and distribution of sequence groups in Fig. 1. (PDF) [file pone.0117499.s001.pdf]

Figure Abbreviation, Reference Number, Sequence Identifier, And Sequence Descriptions
N = not included in Figure 1; I, II, III, and IV are Figure 1 sequence groupings
Numbers before sequence are: beginning sequence number - ending sequence number (as reported in PDB file) and number of amino acids in the row.

```
1A4G-IB  [9]       1A4G.pdb         neuraminidase [Influenza B virus B/Beijing/1/87] complexed with zanamivir
                   1234567890123456789012345678901234567890123456789012345678901234567890123456789012345678901234567890
I      76-177 102  EPEWTYPRLSCQGSTFQKALLISPHRFGEARGNSAPLIIREPFIACGPKECKHFALTHYAAQPGGYYNGTREDRNKLRHLISVKLGKIPTVENSIFHMAAWS
II    178-278 101  GSACHDGREWTYIGVDGPDSNALIKIKYGEAYTDTYHSYANNILRTQESACNCIGGDCYLMITDGSASGISKCRFLKIREGRIIKEIFPTGRVEHTEECTC
III   279-373  95  GFASNKTIECACRDNSYTAKRPFVKLNVETDTAEIRLMCTETYLDTPRPDDGSITGPCESNGDKGRGGIKGGFVHQRMASKIGRWYSRTMSKTER
IV    374-465  92  MGMELYVRYDGDPWTDSDALAHSGVMVSMKEPGWYSFGFEIKDKKCDVPCIGIEMVHDGGKKTWHSAATAIYCLMGSGQLLWDTVTGVDMAL

3209-01 [11]       ADR32096.1       neuraminidase [Influenza A virus (A/Lyon/1364/2007(H1N1))]
                   1234567890123456789012345678901234567890123456789012345678901234567890123456789012345678901234567890
N        1-60  60  MLQIGNIISIWASHSIQTGSQNNTGICNQRIITYENSTWVNHTYVNINNTNVVAGEDKTS
I      61-158  98  VTLAGNSSLCSISGWAIYTKDNSIRIGSKGDVFVIREPFISCSHLECRTFFLTQGALLNDKHSNGTVKDRSPYRALMSCPLGEAPSPYNSKFESVAWS
II    159-259 101  ASACHDGMGWLTIGISGPDNGAVAVLKYNGIITGTIKSWKKQILRTQESECVCMNGSCFTIMTDGPSNKAASYKIFKIEKGKVTKSIELNAPNFHYEECSC
III   260-350  91  YPDTGIVMCVCRDNWHGSNRPWVSFNQNLDYQIGYICSGVFGDNPRPEDGEGSCNPVTVDGANGVKGFSYKYDNGVWIGRTKSNRLRKGFE
IV    351-435  85  MIWDPNGWTNTDSDFSVKQDVVAITDWSGYSGSFVQHPELTGLDCIRPCFWVELVRGLPRENTTIWTSGSSISFCGVNSDTANWS

4790-01 [12]       ACJ47909.1       neuraminidase [Influenza A virus (A/environment/Qinghai/1/2008(H5N1))]
                   1234567890123456789012345678901234567890123456789012345678901234567890123456789012345678901234567890
N        1-62  62  MNPNQKIITIGSICMVIGIVSLMLQIGNIISIWVSHSIQTGNQHQAEPISNTNFLADKAVAS
I      63-159  98  VTLAGNSSLCPISGWAVHSKDNGKRIGSKGDVFVIREPFISCSHLECRTFFLTQGALLNDKHSNGTVKDRSPHRTLMSCPVGEAPSPYNSRFESVAWS
II    160-260 101  ASACHDGTSWLTIGISGPDNGAVAVLKYNGIITDTIKSWRNNILRTQESECACVNGSCFTVMTDGPSNGQASYKIFKMEKGKVVKSVELDAPNYHYEECSC
III   261-350  91  YPDAGEITCVCRDNWHGSNRPWVSFNQNLEYQIGYICSGVFGDNPRPNDGTGSCGPVSPNGAYGVKGFSFKYGNGVWIGRTKSINSRSGFE
IV    351-449  97  MIWDPNGWTGTDSSFSVKQDIVAITDWSGYSGSFVQHPELTGLDCIRPCFWVELIRGRPKESTIWTSGSSISFCGVNSDTVGWSWPDGAELPFTIDK

5091-01 [13]       AF509109.2       neuraminidase [Influenza A virus (A/Chicken/Hong Kong/873.3/01 (H5N1))]
                   1234567890123456789012345678901234567890123456789012345678901234567890123456789012345678901234567890
N        1-82  82  MNPNQKIITIGSICMVIGIVSLMLQIGNIISIWVSHSIQTGNQHQAEPCNQSIITYENNTWVNQTYVNISNTNFLTEKAVAS
I      83-180  98  VTLAGNSSLCPISGWAVYSKDNGIRIGSKGDVFVIREPFISCSHLECRTFFLTQGALLNDKHSNGTVKDRSPYRTLMSCPVGEAPSPYNSRFESVAWS
II    181-281 101  ASACHDGTSWLTIGISGPDNGAVAVLKYNGIITDTIKSWRNNILRTQESECACVNGSCFTVMTDGPSNGQASYKIFKIEKGKVVKSVELNAPNYHYEECSC
III   282-372  91  YPDAGEITCVCRDNWHGSNRPWVSFNQNLEYQIGYICSGVFGDNPRPNDGTGSCGPVSPNGAYGIKGFSFKYGNGVWIGRTKSTNSRSGFE
IV    373-469  97  MIWDPNGWTGTDSNFSVKQDIVAITDWSGYSGSFVQHPELTGVDCIRPCFWVELIRGRPKESTIWTSGSSISFCGVNSDTVGWSWPDGAELPFTIDK

5343-01 [14]       AEV53435.1       neuraminidase [Influenza A virus (A/Fukushima/09FY004/2009(H1N1))]
                   1234567890123456789012345678901234567890123456789012345678901234567890123456789012345678901234567890
N        1-92  92  MNPNQKIITIGSVCMTIGMANLILQIGNIISIWISHSIQLGNQNQIETCNQSVITYENNTWVNQTYVNISNTNFAAGQSVVS
I      93-190  98  VKLAGNSSLCPVSGWAIYSKDNSIRIGSKGDVFVIREPFISCSPLECRTFFLTQGALLNDKHSNGTIKDRSPYRTLMSCPIGEVPSPYNSRFESVAWS
II    191-291 101  ASACHDGINWLTIGISGPDNGAVAVLKYNGIITDTIKSWRNNILRTQESECACVNGSCFTVMTDGPSDGQASYKIFRIEKGKIVKSVEMNAPNYHYEECSC
III   292-382  91  YPDSSEITCVCRDNWHGSNRPWVSFNQNLEYQIGYICSGIFGDNPRPNDKTGSCGPVSSNGANGVKGFSFKYGNGVWIGRTKSISSRNGFE
IV    383-479  97  MIWDPNGWTGTDNNFSIKQDIVGINEWSGYSGSFVQHPELTGLDCIRPCFWVELIRGRPKENTIWTSGSSISFCGVNSDTVGWSWPDGAELPFTIDK

5971-02 [15]       ADG59718.1       neuraminidase [Influenza A virus (A/El Salvador/2-Q226L/1957(H2N2))]
                   1234567890123456789012345678901234567890123456789012345678901234567890123456789012345678901234567890
N        1-82  82  MNPNQKIITIGSVSLTIATACLLMQIAILAATVTLHFKQHECDSPASNQVMPCEPIIIERNITEIVYLNNTTIEKEICPEVV
I      83-179  97  EYRNWSKPQCQITGFAPFSKDNSIRLSAGGDIWVTREPYVSCDPGKCYQFALGQGTTLDNKHSNGTIHDRIPHRTLLMNELGVPFHLGTKQVCVAWS
II    180-280 101  SSSCHDGKAWLHVCVTGDDRNATASFIYDGRLVDSIGSWSQNILRTQESECVCINGTCTVVMTDGSASGRADTRILFIKEGKIVHISPLSGSAQHIEECSC
III   281-375  95  YPRYPDVRCICRDNWKGSNRPVIDINMEDYSIDSSYVCSGLVGDTPRNDDSSSNSNCRDPNNERGNPGVKGWAFDNGDDVWMGRTINKDSRSGYE
IV    376-469  94  TFKVIGGWSTPNSKSQVNRQVIVDNNNWSGYSGIFSVEGKSCINRCFYVELIRGRPQETRVWWTSNSIVVFCGTSGTYGTGSWPDGANINFMPI
```

Figure Abbreviation, Reference Number, Sequence Identifier, And Sequence Descriptions
N = not included in Figure 1; I, II, III, and IV are Figure 1 sequence groupings
Numbers before sequence are: beginning sequence number - ending sequence number (as reported in PDB file) and number of amino acids in the row.

```
8342-02 [16]       AGW83423.1       neuraminidase [Influenza A virus (A/Djibouti/N09200/2009(H3N2))]
                   1234567890123456789012345678901234567890123456789012345678901234567890123456789012345678901234567890
N        1-82  82  MNPNQKIITIGSVSLTISTICFFMQTAILITTVTLHFKQCEFNSPPNNQVMLCEPTIIERNITEIVYLTNTTIEKEICPKLA
I       83-179  97 EYRNWSKPQCDITGFAPFSKDNSIRLSAGGDIWVTREPYVSCDPDKCYQFALGQGTTLNNVHSNNTVRDRTPYRTLLMNELGVPFHLGTKQVCIAWS
II     180-280 101 SSSCHDGKAWLHVCITGDDKNATASFIYNGRLVDSVVSWSKEILRTQESECVCINGTCTVVMTDGSASGKADTKILFIEEGKIVHTSTLSGSAQHVEECSC
III    281-375  95 YPRYPGVRCVCRDNWKGSNRPIVDINIKDHSIVSSYVCSGLVGDTPRKNDSSSSHCLDPNNEEGGHGVKGWAFDDGNDVWMGRTISEKSRFGYE
IV     376-469  94 TFKVIEGWSNPKSKLQINRQVIVDRGNRSGYSGIFSVEGKSCINRCFYVELIRGRKEETEVLWTSNSIVVFCGTSGTYGTGSWPDGADINLMPI
```

```
6202-03 [17]       AAO62026.1       neuraminidase [Influenza A virus (A/Goose/HonGKonG/27404/78(H5N3))]
                   1234567890123456789012345678901234567890123456789012345678901234567890123456789012345678901234567890
N        1-92  92  MNPNQKIITIGVVNTTLSTIALLIGVGNLIFNTVIHEKIGDHQTVVYPTITTPVVPNCSDTIITYNNTVINNITTTIITETE
I       93-180  98 RHFKPSLPLCPFRGFFPFHKDNAIRLGENKDVIVTREPYVSCDNDNCWSFALAQGALLGTKHSNGTIKDRTPYRSLIRFPIGTAPVLGNYKEICVAWS
II     181-282 102 SSSCFDGKEWMHVCMTGNDNDASAQIIYAGKMTDSIKSWRRDILRTQESECQCIDGTCIVVVTDGPAANSADHRIYWIRRGKVIKYENVPKTKIQHLEECSC
III    283-374  92 YVDTDVYCICRDNWKGSNRPWMRINNETILETGYVCSKFHSDTPRPADPSTVSCDSPSNVNGGPGVKGFGFKAGNDVWLGRTVSTSGRSGFE
IV     375-469  95 IIKVTEGWINSPNHAKSLTQTLVSNNDWSGYSGSFIIENNGCFQPCFYIELIRGRPNKNDDVSWTSNSIVTFCGLDNEPGSGNWPDGSNIGFMPK
```

```
1150-09 [19]       AHA11501.1       neuraminidase [Influenza A virus (A/ZhejianG/DTID-ZJU10/2013(H7N9))]
                   1234567890123456789012345678901234567890123456789012345678901234567890123456789012345678901234567890
N        1-78  78  MNPNQKILCTSATAIIIGAIAVLIGIANLGLNIGLHLKPGCNCSHSQPETTNTSQTIINNYYNETNITNIQMEERTSR
I       79-176  98 NFNNLTKGLCTINSWHIYGKDNAVRIGESSDVLVTREPYVSCDPDECRFYALSQGTTIRGKHSNGTIHDRSQYRALISWPLSSPPTVYNSRVECIGWS
II     177-277 101 STSCHDGKSRMSICISGPNNNASAVVWYNRRPVAEINTWARNILRTQESECVCHNGVCPIVFTDGSATGPADTRIYYFKEGKILEWESLTGTAKHIEECSC
III    278-371  94 YGERTGITCTCRDNWQGSNRPVIQIDPVAMTHTSQYICSPVLTDTPRPNDPNIGKCNDPYPGNDNNGVKGFSYLDGANTWLGRTISTASRSGYE
IV     372-465  94 MLKVPNALTDDRSKPIQGQTIVLNADWSGYSGSFMDYWAEGDCYRACFYVELIRGRPKEDKVWWTSNSIVSMCSSTEFLGQWNWPDGARIEYFL
```

```
6207-06 [18]       AAO62070.1       neuraminidase [Influenza A virus (A/quail/NanchanG/4-034/2000(H4N6))]
                   1234567890123456789012345678901234567890123456789012345678901234567890123456789012345678901234567890
N        1-92  92  MNPNQKIICISATGMTLSVVSLLIGIANLGLNIGLHYKMGDTPDVNIPNMNETNSTTTIINNHTQNNFTNITNIIVNKNEEG
I       93-190  98 TFLNLTKPLCEVNSWHILSKDNAIRIGEDAHILVTREPYLSCDPQGCRMFALSQGTTLRGRHANGTIHDRSPFRALISWEMGQAPSPYNVRVECIGWS
II     191-291 101 STSCHDGISRMSICMSGANNNASAVVWYGGRPVTEIPSWAGNILRTQESECVCHKGICPVVMTDGPANNRAATKIIYFKEGKIQKIEELAGNTQHIEECSC
III    292-386  95 YGAVGVIKCICRDNWKGANRPVITIDPEMMTHTSKYLCSKILTDTSRPNDPTNGNCDAPITGGSPDPGVKGFAFLDRENSWLGRTISKDSRSGYE
IV     387-480  94 MLKVPNAETDTQSGPISHQVIVNNQNWSGYSGAFIDYWANKECFNPCFYVELIRGRPKESSVLWTSNSIVALCGSKERLGSWSWHDGAEIIYFK
```

```
1W1X-06  [8]       1W1X.pdb         neuraminidase from english duck subtype N6 complexed with 30 mm sialic acid (NANA, NEU5AC)
                   1234567890123456789012345678901234567890123456789012345678901234567890123456789012345678901234567890
N          88   1  R
I       89-186  98 TFLNLTKPLCEVNSWHILSKDNAIRIGEDAHILVTREPYLSCDPQGCRMFALSQGTTLRGRHANGTIHDRSPFRALISWEMGQAPSPYNTRVECIGWS
II     187-287 101 STSCHDGMSRMSICMSGPNNNASAVVWYGGRPITEIPSWAGNILRTQESECVCHKGVCPVVMTDGPANNRAATKIIYFKEGKIQKIEELAGNAQHIEECSC
III    288-382  95 YGAGGVIKCICRDNWKGANRPVITIDPEMMTHTSKYLCSKVLTDTSRPNDPTNGNCDAPITGGSPDPGVKGFAFLDGENSWLGRTISKDSRSGYE
IV     383-476  94 MLKVPNAETDIQSGPISNQVIVNNQNWSGYSGAFIDYWANKECFNPCFYVELIRGRPKESSVLWTSNSIVALCGSKKRLGSWSWHDGAEIIYFE
```

```
4FVK-10  [5]       4FVK.pdb         neuraminidase-like molecule N10 derived from bat influenza A virus fragment
                   1234567890123456789012345678901234567890123456789012345678901234567890123456789012345678901234567890
N       74-82   9  GSPSRSPEF
I       83-179  95 FYWRAKSQMCEVKGWVPTHRGFPWGPELPGDLILSRRAYVSCDLTSCFKFFIAYGLSANQHLLNTSMEWEESLYKTPIGSASTLSTSEMILPGRS
II     180-280 101 SSACFDGLKWTVLVANGRDRNSFIMIKYGEEVTDTFSASRGGPLRLPNSECICIEGSCFVIVSDGPNVNQSVHRIYELQNGTVQRWKQLNTTGINFEYSTC
III    281-374  90 YTINNLIKCTGTNLWNDAKRPLLRFTKELNYQIVEPCNGAPTDFPRGGLTTPSCKMAQEKGEGGIQGFILDEKPAWTSKTKAESSQNGFV
IV     375-442  81 LEQIPNGIESEGTVSLSYELFSNKRTGRSGFFQPKGDLISGCQRICFWLEIEDQTVGLGMIQELSTFCGINSPVQNINWDS
```

Figure Abbreviation, Reference Number, Sequence Identifier, And Sequence Descriptions
N = not included in Figure 1; I, II, III, and IV are Figure 1 sequence groupings
Numbers before sequence are: beginning sequence number - ending sequence number (as reported in PDB file) and number of amino acids in the row.

```
1259-11 [20]       CY125947.1       neuraminidase-like protein [Influenza A virus (A/flat-faced bat/Peru/033/2010(H18N11))]
                   1234567890123456789012345678901234567890123456789012345678901234567890123456789012345678901234567890
N         1-84  84 MSFQTSTCLLIVSLICGILTVCLQVLLPFILIWTNTEPNYSCECPAPNISLSCPNGTSVTYDSKNITENSFYSSTTNYLSPVIA
I       85-179  95 TPLVLGENLCSINGWVPTYRGEGTTGKIPDEQMLTRQNFVSCSDKECRRFFVSMGYGTTTNFADLIVSEQMNVYSVKLGDPPTPDKLKFEAVGWS
II     180-278  98 ASSCHDGFQWTVLSVAGDGFVSILYGGIITDTIHPTNGGPLRTQASSCICNDGTCYTIIADGTTYTASSHRLYRLVNGTSAGWKALDTTGFNFEFPTC
III    279-368  90 YYTSGKVKCTGTNLWNDAKRPFLEFDQSFTYTFKEPCLGFLGDTPRGIDTTNYCDKTTTEGEGGIQGFMIEGSNSWIGRIINPGSKKGFE
IV     369-448  80 IYKFLGTLFSVQTVGNRNYQLLSNSTIGRSGLYQPAYESRDCQELCFWIEIAATTKAGLSSNDLITFCGTGGSMPDVNWG

4K3Y-11  [7]       4K3Y.pdb         neuraminidase-like protein of A/flat-faced bat/Peru/033/2010(H18N11)
                   1234567890123456789012345678901234567890123456789012345678901234567890123456789012345678901234567890
N           82   1 A
I       83-179  95 TPLVLGENLCSINGWVPTYRGEGTTGKIPDEQMLTRQNFVSCSDKECRRFFVSMGYGTTTNFADLIVSEQMNVYSVKLGDPPTPDKLKFEAVGWS
II     180-280  98 ASSCHDGFQWTVLSVAGDGFVSILYGGIITDTIHPTNGGPLRTQASSCICNDGTCYTIIADGTTYTASSHRLYRLVNGTSAGWKALDTTGFNFEFPTC
III    281-375  90 YYTSGKVKCTGTNLWNDAKRPFLEFDQSFTYTFKEPCLGFLGDTPRGIDTTNYCDKTTTEGEGGIQGFMIEGSNSWIGRIINPGSKKGFE
IV     376-459  80 IYKFLGTLFSVQTVGNRNYQLLSNSTIGRSGLYQPAYESRDCQELCFWIEIAATTKAGLSSNDLITFCGTGGSMPDVNWG

SPN     [10]       3H72.pdb         streptococcus pneumoniae D39 neuraminidase A precursor (complexed with NANA)
                   1234567890123456789012345678901234567890123456789012345678901234567890123456789012345678901234567890
N      317-319   3 LPE
I      320-417  98 GAALTEKTDIFESGRNGKPNKDGIKSYRIPALLKTDKGTLIAGADERRLHSSDWGDIGMVIRRSEDNGKTWGDRVTITNLRDNPKASDPSIGSPVNID
II     418-650 248 MVLVQDPETKRIFSIYDMFPEGKGIFGMSSQKEEAYKKIDGKTYQILYREGEKGAYTIRENGTVYTPDGKATDYRVVVDPVKPAYSDKGDLYKGNQLLGN
                   IYFTTNKTSPFRIAKDSYLWMSYSDDDGKTWSAPQDITPMVKADWMKFLGVGPGTGIVLRNGPHKGRILIPVYTTNNVSHLNGSQSSRIIYSDDHGKTWH
                   AGEAVNDNRQVDGQKIHSSTMNNRRAQNTESTV
III    651-724  74 VQLNNGDVKLFMRGLTGDLQVATSKDGGVTWEKDIKRYPQVKDVYVQMSAIHTMHEGKEYIILSNAGGPKRENGM
IV     725-793  69 VHLARVEENGELTWLKHNPIQKGEFAYNSLQELGNGEYGILYEHTEKGQNAYTLSFRKFNWDFLSKDL

2G9H    [22]       2G9H.pdb         staphylococcal enterotoxin I (SEI) chain D (complexed with a human MHC class II molecule)
                   1234567890123456789012345678901234567890123456789012345678901234567890123456789012345678901234567890
         1-218 218 QGDIGVGNLRNFYTKHDYIDLKGLIDKNLPSANQLEFSTGINDLISESNNWDEISKFKGKKLDIFGIDYNGPCKSKYMYGGATLSGQYLNSARKIPINLW
                   VNGKHKTISTDKISTNKKLVTAQEIDVKLRRYLQEEYNIYGHNSTGKGKEYGYKSKFYSGFNKGKVLFHLNDEKSFSYDLFYTGDGVPVSFLKIYEDNKI
                   IESEKFHLDVEISYVDSN

3RKD    [23]       3RKD.pdb         hepatitis E virus E2S domain genotype I (complexed with a neutralizing antibody)
                   1234567890123456789012345678901234567890123456789012345678901234567890123456789012345678901234567890
       448-604 147 SRPFSVLRANDVLWLSLTAAEYDQSTYGSSTGPVYVSDSVTLVNVATGAQAVARSLDWTKVTLDGRPLSTIQQHSKTFFVLPLRGKLSFWEAGTTKAGYP
                   YNYNTTASDQLLVENAAGHRVAISTYTTSLGAGPVSISAVAVLAPP

3SCK    [24]       3SCK.pdb         spike protein receptor-binding domain from a predicted SARS coronavirus civet strain
                                    (complexed with human-civet chimeric receptor ACE2 fragment)
                   1234567890123456789012345678901234567890123456789012345678901234567890123456789012345678901234567890
       324-502 179 PFGEVFNATKFPSVYAWERKKISNCVADYSVLYNSTFFSTFKCYGVSATKLNDLCFSNVYADSFVVKGDDVRQIAPGQTGVIADYNYKLPDDFMGCVLAW
                   NTRNIDATSTGNYNYKYRYLRHGKLRPFERDISNVPFSPDGKPCTPPAPNCYWPLRGYGFYTTTGIGYQPYRVVVLSFE

2ABX    [25]       2ABX.pdb         alpha-bungarotoxin complexed to acetylcholine receptor
                   1234567890123456789012345678901234567890123456789012345678901234567890123456789012345678901234567890
          1-74  74 IVCHTTATIPSSAVTCPPGENLCYRKMWCDAFCSSRGKVVELGCAATCPSKKPYEEVTCCSTDKCNHPPKRQPG

2KS9    [26]       2KS9.pdb         substance P in water (complexed with NK1R, substance-P receptor tachykinin receptor 1)
                   1234567890123456789012345678901234567890123456789012345678901234567890123456789012345678901234567890
       365-375  11 RPKPQQFFGLM
```

Figure Abbreviation, Reference Number, Sequence Identifier, And Sequence Descriptions
N = not included in Figure 1; I, II, III, and IV are Figure 1 sequence groupings
Numbers before sequence are: beginning sequence number - ending sequence number (as reported in PDB file) and number of amino acids in the row.

```
1DLL      [29]      1DLL.pdb  THE HC FRAGMENT OF TETANUS TOXIN  RECEPTOR BINDING FRAGMENT HC FROM CLOSTRIDIUM TETANI
                    1234567890123456789012345678901234567890123456789012345678901234567890123456789012345678901234567890
      693-1315      EDIDVILKKSTILNLDINNDIISDISGFNSSVITYPDAQLVPGINGKAIHLVNNESSEVIVHKAMDIEYNDMFNNFTVSFWLRVPKVSASHLEQYGTNEY
                    SIISSMKKHSLSIGSGWSVSLKGNNLIWTLKDSAGEVRQITFRDLPDKFNAYLANKWVFITITNDRLSSANLYINGVLMGSAEITGLGAIREDNNITLKL
                    DRCNNNNQYVSIDKFRIFCKALNPKEIEKLYTSYLSITFLRDFWGNPLRYDTEYYLIPVASSSKDVQLKNITDYMYLTNAPSYTNGKLNIYYRRLYNGLK
                    FIIKRYTPNNEIDSFVKSGDFIKLYVSYNNNEHIVGYPKDGNAFNNLDRILRVGYNAPGIPLYKKMEAVKLRDLKTYSVQLKLYDDKNASLGLVGTHNGQ
                    IGNDPNRDILIASNWYFNHLKDKILGCDWYFVPTDEGWTND

3ZUQ      [28]      3ZUQ.pdb  STRUCTURE OF AN ENGINEERED BOTULINUM NEUROTOXIN TYPE B FROM CLOSTRIDIUM BOTULINUM
                    1234567890123456789012345678901234567890123456789012345678901234567890123456789012345678901234567890
         1-892      MPVTINNFNYNDPIDNNNIIMMEPPFARGTGRYYKAFKITDRIWIIPERYTFGYKPEDFNKSSGIFNRDVCEYYDPDYLNTNDKKNIFLQTMIKLFNRIK
                    SKPLGEKLLEMIINGIPYLGDRRVPLEEFNTNIASVTVNKLISNPGEVERKKGIFANLIIFGPGPVLNENETIDIGIQNHFASREGFGGIMQMKFCPEYV
                    SVFNNVQENKGASIFNRRGYFSDPALILMHELIHVLHGLYGIKVDDLPIVPNEKKFFMQSTDAIQAEELYTFGGQDPSIITPSTDKSIYDKVLQNFRGIV
                    DRLNKVLVCISDPNINIYKNKFKDKYKFVEDSEGKYSIDVESFDKLYKSLMFGFTETNIAENYKIKTRASYFSDSLPPVKIKNLLDNEIYTIEEGFNI
                    SDKDMEKEYRGQNKAINKQAYEEISKEHLAVYKIQMCVDGGGGSGGGGSGGGGSAIEGRAGGGGSGGGGSGGGGSALVLQCIDVDNEDLFFIADKNSFSD
                    DLSKNERIEYNTQSNYIENDFPINELILDTDLISKIELPSENTESLTDFNVDVPVYEKQPAIKKIFTDENTIFQYLYSQTFPLDIRDISLTSSFDDALLF
                    SNKVYSFFSMDYIKTANKVVEAGLFAGWVKQIVNDFVIEANKSNTMDKIADISLIVPYIGLALNVGNETAKGNFENAFEIAGASILLEFIPELLIPVVGA
                    FLLESYIDNKNKIIKTIDNALTKRNEKWSDMYGLIVAQWLSTVNTQFYTIKEGMYKALNYQAQALEEIIKYRYNIYSEKEKSNINIDFNDINSKLNEGIN
                    QAIDNINNFINGCSVSYLMKKMIPLAVEKLLDFDNTLKKNLLNYIDENKLYLIGSAEYEKSKVNKYLKTIMPFDLSIYTNDTILIEMFNKYNSLEALASG
                    HHHHHH

1YQY      [27]      1YQY.pdb STRUCTURE OF B. ANTHRAX LETHAL FACTOR
                    1234567890123456789012345678901234567890123456789012345678901234567890123456789012345678901234567890
       257-780      GSKDPGMLSRYEKWEKIKQHYQHWSDSLSEEGRGLLKKLQIPIEPKKDDIIHSLSQEEKELLKRIQIDSSDFLSTEEKEFLKKLQIDIRDSLSEEEKELL
                    NRIQVDSSNPLSEKEKEFLKKLKLDIQPYDINQRLQDTGGLIDSPSINLDVRKQYKRDIQNIDALLHQSIGSTLYNKIYLYENMNINNLTATLGAADLVD
                    STDNTKINRGIFNEFKKNFKYSISSNYMIVDINERPALDNERLKWRIQLSPDTRAGYLENGKLILQRNIGLEIKDVQIIKQSEKEYIRIDAKVVPKSKID
                    TKIQEAQLNINQEWNKALGLPKYTKLITFNVHNRYASNIVESAYLILNEWKNNIQSDLIKKVTNYLVDGNGRFVFTDITLPNIAEQYTHQDEIYEQVHSK
                    GLYVPESRSILLHGPSKGVELRNDSEGFIHEFGHAVDDYAGYLLDKNQSDLVTNSKKFIDIFKEEGSNLTSYGRTNEAEFFAEAFRLMHSTDHAERLKVQ
                    KNAPKTFQFINDQIKFIINSLVPR
```
